# Major Families of Multiresistant Plasmids from Geographically and Epidemiologically Diverse Staphylococci

**DOI:** 10.1534/g3.111.000760

**Published:** 2011-12-01

**Authors:** Julia E. S. Shearer, Joy Wireman, Jessica Hostetler, Heather Forberger, Jon Borman, John Gill, Susan Sanchez, Alexander Mankin, Jacqueline LaMarre, Jodi A. Lindsay, Kenneth Bayles, Ainsley Nicholson, Frances O’Brien, Slade O. Jensen, Neville Firth, Ronald A. Skurray, Anne O. Summers

**Affiliations:** * Department of Microbiology; ‡ Department of Infectious Diseases, College of Veterinary Medicine, University of Georgia, Athens, Georgia 30602; † J. Craig Venter Institute, Rockville, Maryland 20850; § Center for Pharmaceutical Biotechnology, University of Illinois, Chicago, Illinois 60607; ** Department of Cellular and Molecular Medicine, St. George’s, University of London, London SW17 0RE, United Kingdom; †† Department of Pathology and Microbiology, University of Nebraska Medical Center, Omaha, Nebraska 68198; ‡‡ Division of Healthcare Quality Promotion, Centers for Disease Control and Prevention, Atlanta, Georgia 30333; §§ School of Biomedical Sciences, Curtin University of Technology, Perth 6000, Western Australia; *** School of Medicine, University of Western Sydney, Sydney 2751, Australia; ††† School of Biological Sciences, University of Sydney, Sydney 2006, Australia

**Keywords:** plasmid, resistance, mobile element genomics, MRSA, horizontal gene transfer

## Abstract

Staphylococci are increasingly aggressive human pathogens suggesting that active evolution is spreading novel virulence and resistance phenotypes. Large staphylococcal plasmids commonly carry antibiotic resistances and virulence loci, but relatively few have been completely sequenced. We determined the plasmid content of 280 staphylococci isolated in diverse geographical regions from the 1940s to the 2000s and found that 79% of strains carried at least one large plasmid >20 kb and that 75% of these large plasmids were 20–30 kb. Using restriction fragment length polymorphism (RFLP) analysis, we grouped 43% of all large plasmids into three major families, showing remarkably conserved intercontinental spread of multiresistant staphylococcal plasmids over seven decades. In total, we sequenced 93 complete and 57 partial staphylococcal plasmids ranging in size from 1.3 kb to 64.9 kb, tripling the number of complete sequences for staphylococcal plasmids >20 kb in the NCBI RefSeq database. These plasmids typically carried multiple antimicrobial and metal resistances and virulence genes, transposases and recombinases. Remarkably, plasmids within each of the three main families were >98% identical, apart from insertions and deletions, despite being isolated from strains decades apart and on different continents. This suggests enormous selective pressure has optimized the content of certain plasmids despite their large size and complex organization.

Staphylococci are common commensals and opportunistic pathogens mainly found on the skin and in the nose of humans and also in domestic and companion animals (Epstein *et al.* 2009; Lindsay 2010; Middleton *et al.* 2005; Rubin *et al.* 2011; Rutland *et al.* 2009; Spohr *et al.* 2011; Sung *et al.* 2008; Van Duijkeren *et al.* 2011; Vanderhaeghen *et al.* 2011; Walther *et al.* 2009; Wassenberg *et al.* 2011). *Staphylococcus aureus*, carried by ∼30% of humans in developed countries (Lindsay and Holden 2004; Plata *et al.* 2009), is a leading cause of healthcare-associated (HA) infections and is increasingly responsible for life-threatening community-acquired (CA) infections in otherwise healthy persons (Diekema *et al.* 2001; Lindsay and Holden 2004, 2006; Navarro *et al.* 2008; Plata *et al.* 2009). *S. aureus* is the number one cause of bloodstream, skin, and lower respiratory infections (Diekema *et al.* 2001; Goetghebeur *et al.* 2007; Plata *et al.* 2009), and multiantibiotic resistances common to HA staphylococci are now increasingly present in the community strains (Lindsay and Holden 2006; McDougal *et al.* 2010; Navarro *et al.* 2008). Coagulase-negative staphylococci (CNS; *e.g.* *Staphylococcus epidermidis*) are also a major cause of HA bloodstream infections, and oxacillin resistance and methicillin resistance are found in over 70% of strains (Diekema *et al.* 2001).

Analyses of *S. aureus* complete genomes revealed that most virulence factors and antibiotic resistance genes are carried on mobile genetic elements (MGE) (Baba *et al.* 2002; Feng *et al.* 2008; Highlander *et al.* 2007; Holden *et al.* 2004; Omoe *et al.* 2003) such as pathogenicity islands, chromosomal cassettes, transposable elements, bacteriophages, and plasmids (Lindsay and Holden 2004, 2006; Lindsay 2010; Malachowa and Deleo 2010; Novick 2003). Thus, understanding the MGEs in staphylococci is critical to controlling dissemination of these virulence factors that markedly increase the hazard of these pathogens.

Multilocus sequence typing (MLST) of methicillin-resistant *Staphylococcus aureus* (MRSA) strains has shown that outbreaks are caused by relatively few clonal complexes or by strains with closely related genotypes (Diekema *et al.* 2001; Enright *et al.* 2002; Feil *et al.* 2003; Feil and Enright 2004; Feng *et al.* 2008; Highlander *et al.* 2007; Lindsay and Holden 2004, 2006). The success of these clonal complexes as pathogens may partially be explained by their enhanced ability to receive MGEs by horizontal gene transfer (HGT). Type I (Sung and Lindsay 2007; Waldron and Lindsay 2006) and type III–like (Corvaglia *et al.* 2010) restriction modification systems in *S. aureus* provide natural barriers to HGT. Strains with defective restriction modification systems acquire MGEs at higher frequencies and have greater potential to become “superbugs” by accumulating virulence factors and resistance genes (Corvaglia *et al.* 2010; Sung and Lindsay 2007; Waldron and Lindsay 2006).

Staphylococcal plasmids carry resistances to antibiotics, metals, antiseptics, and disinfectants, as well as virulence genes, such as enterotoxins (Bayles and Iandolo 1989; Omoe *et al.* 2003) and exfoliative toxins (Jackson and Iandolo 1986; Yamaguchi *et al.* 2001). Plasmids in staphylococci may be horizontally transferred through conjugation, mobilization, and/or transduction (Apisiridej *et al.* 1997; Berg *et al.* 1998; Francia *et al.* 2004; Lindsay and Holden 2004; Malachowa and Deleo 2010; Smith and Thomas 2004; Udo and Grubb 2001; Varella Coelho *et al.* 2009). Staphylococci also carry virulence plasmids originating from *Bacillus* (Gill *et al.* 2005) and *Enterococcus* (Clewell *et al.* 1985; Noble *et al.* 1992; Périchon and Courvalin 2009; Sung and Lindsay 2007; Weigel *et al.* 2003).

Staphylococcal plasmids are taxonomically grouped by replication mechanism and conjugation ability: the small, usually <5 kb, rolling-circle replicating (RCR) plasmids; the larger theta-replicating plasmids, which are subdivided into the pSK41-like conjugative plasmids; and the nonconjugative antimicrobial and metal resistance plasmids (Berg *et al.* 1998; Firth *et al.* 2000; Firth and Skurray 2006; Khan 1997; Malachowa and Deleo 2010; Novick 1989). The small RCR plasmids often carry a single antibiotic resistance gene (Khan 1997) that is transferred by transducing phages, mobilized by conjugative plasmids, or can form unresolved cointegrates with conjugative or mobilizable plasmids, arising from replicative transposition by IS257 or homologous recombination between IS257 elements (Berg *et al.* 1998; Leelaporn *et al.* 1996; Smith and Thomas 2004; Varella Coelho *et al.* 2009). The theta-replicating nonconjugative plasmids can also be transferred by mobilization or transduction (Apisiridej *et al.* 1997; Francia *et al.* 2004; Lindsay and Holden 2006; Malachowa and Deleo 2010; Smillie *et al.* 2010). As of 2010, the NCBI RefSeq database had 102 complete staphylococcal plasmid sequences, but only 29 (28%) of them were >20 kb and only 15% were >30 kb and, thus, large enough to encode conjugation machinery. Of the latter group, only 6% had annotated conjugative transfer loci (McDougal *et al.* 2010). Conjugative plasmids spread readily among *Staphylococcus* strains and to and from other genera such as *Enterococcus* (Lindsay 2010; Malachowa and Deleo 2010; Périchon and Courvalin 2009; Zhu *et al.* 2008), and they are implicated in spreading virulence loci and vancomycin resistance among clinical strains (Périchon and Courvalin 2009; Zhu *et al.* 2008). Multiresistance plasmids between 20 and 30 kb are common in staphylococci from several continents (Baba *et al.* 2002; Bayles and Iandolo 1989; Highlander *et al.* 2007; Holden *et al.* 2004; Shalita *et al.* 1980; Toh *et al.* 2007; Zuccarelli *et al.* 1990). Although unlikely to be conjugative, these 20–30 kb multiresistance virulence plasmids can be transferred by mobilization or by the generalized transducing phages prevalent among *S. aureus* strains (Lindsay and Holden 2004, 2006; Lindsay 2010).

We undertook the work reported here to identify the plasmid composition of naturally occurring isolates of staphylococci and found considerably more large plasmids than previously recorded. We used this opportunity to examine archived strains from the mid-twentieth century known to contain large plasmids. We screened 280 strains of staphylococci collected from 1946 to 2007 in diverse geographical regions to determine the number, size, and restriction type of plasmids they carried (aka the plasmid profile of each strain). We chose 100 strains containing distinct plasmids >20 kb for sequencing to increase the representation of large staphylococcal plasmid sequences available. The 93 complete new plasmid sequences included 59 plasmids >20 kb, tripling the number of large staphylococcal plasmid sequences in RefSeq. We also acquired 57 partial plasmid and phage sequences.

## Materials and Methods

### Strain collections

We screened 280 strains of staphylococci from eight geographically and epidemiologically distinct collections (supporting information, Table S1), including 251 *S. aureus*, 14 *S. epidermidis*, 3 *S. lentus*, 2 *S. pseudintermedius*, 2 *S. schleiferi*, and 8 CNS of unspecified species. In addition, we sequenced plasmid DNA from strain CM05, a human clinical isolate from Columbia (Toh *et al.* 2007) and from vancomycin-resistant *Enterococcus faecium* strain 5753c (McDougal *et al.* 2010).

### Isolation and characterization of plasmid DNA

Strains were grown in BHI broth without shaking at 37° for 18–24 hr. Total plasmid DNA was isolated from 1 to 5 ml of culture with the CosMC Prep Kit (Agencourt Biosciences Corp., Beverly, MA), as described (Williams *et al.* 2006). Cell pellets were suspended in 100 μl RE1 buffer containing 200 μg/ml lysostaphin and 6% PEG (MW 8000) and incubated for 5 min at room temperature before proceeding with the CosMC Prep Kit protocol. CosMC Kit preparations were done in duplicate, and pooled plasmid DNA was electrophoresed using 0.5% SeaKem Gold agarose (Cambrex BioScience, Walkersville, MD) 16 cm gels in 1X TAE (40 mM Tris-acetate, 2 mM Na_2_EDTA–2H_2_O) for 15–18 hr at 30–35 V and stained with Sybr Green I (Invitrogen, Carlsbad, CA). Plasmid band sizes were estimated by BacTracker supercoiled DNA ladder (Epicentre, Madison, WI). DNA preparations found initially to have high sheared DNA background were in subsequent preparations treated with lambda exonuclease and RecJ_f_ (New England Biolabs) (Balagurumoorthy *et al.* 2008) and/or Plasmid-safe DNase (Epicentre) prior to electrophoresis. Sixteen plasmids were further purified by electroelution (Williams *et al.* 2006) into 1X TAE followed by TE dialysis. Three plasmids were prepared by QIAprep Spin Miniprep Kit (Qiagen) or Qiagen Midi Kit (*Enterococcus faecium* 5753c), and one plasmid (pCM05) was prepared by CsCl gradient ultracentrifugation.

Plasmid bands were characterized by restriction fragment length polymorphism (RFLP) analysis, and plasmids were assigned a restriction type (RT) according to their *AccI* RFLP pattern; a unique RT number was given to each unique pattern. In-slice restriction digests were performed on gel slices containing individual plasmid bands excised with a razor blade. Gel slices were rocked at 4° for 30 min in 1 ml TE (10 mM Tris-HCl, 1 mM EDTA, pH 8), transferred to 100 μl 1X restriction buffer (New England Biolabs), rocked at 4° for 30 min to 1 hr, transferred to 100 μl fresh 1X restriction buffer with 15–20 units enzyme, and then incubated at 37°, 50–75 rpm for 16–24 hr. Digested gel slices were transferred to 1 ml 1X TBE and rocked at 4° for 30–60 min, sealed with molten agarose into a well of a 16 cm 1.5% agarose gel (medium EEO) (Sigma, Inc., St. Louis, MO), and electrophoresed in 1X TBE for 15–18 hr at 30–35 V. Gels were stained with Sybr Green I (Invitrogen). RFLP patterns with several enzymes guided the choice of those for sequencing and subsequently tested the accuracy of the sequence assemblies.

### Plasmid DNA sequencing

Plasmid DNA was sequenced according to standard high-throughput Sanger protocols at the J. Craig Venter Institute, and data was assembled using the Celera Assembler (Myers *et al.* 2000). Quality control inspections included coverage analysis, BLAST (http://blast.ncbi.nlm.nih.gov) (Zhang *et al.* 2000) of contigs >2000 bases, and visual inspection of mate pairing and scaffolding using Hawkeye (Schatz *et al.* 2007). Chromosomal and/or other contamination were filtered based on perfect BLAST hits to *S. aureus* and *S. epidermidis* references. DNA samples for each strain were assigned a unique SAP (*S. aureus* plasmids) project number. To distinguish multiple plasmids in the same strain, each was assigned the strain SAP number plus A, B, C, or D, starting with complete (closed) plasmid sequences in descending size, followed by partial sequences (Table S2).

### Sequence analysis

Gene calling and annotations were done by P-RAST (http://cgat.mcs.anl.gov/plasmid-rast-dev/FIG/prast.cgi) and/or RAST (http://rast.nmpdr.org) (Aziz *et al.* 2008), which uses mobile element gene names from the ACLAME database (http://aclame.ulb.ac.be) (Leplae *et al.* 2004). Lasergene (DNAstar, Madison, WI) and Gene Construction Kit (Textco Biosoftware, West Lebanon, NH) were used to display sequences and predict restriction sites. Sequence alignments were done with Megalign (DNAstar) using ClustalW and/or with BLAST (http://blast.ncbi.nlm.nih.gov) (Zhang *et al.* 2000), and plasmid genome alignments were done using Mauve (Darling *et al.* 2004). One hundred fifty complete and partial plasmid and phage sequences were submitted to GenBank (http://www.ncbi.nlm.nih.gov) (Table S2). Partial sequences are those with one or more gaps in the sequence, including plasmid and phage fragments (Table S2). Partial sequences with the same plasmid name are fragments of the same plasmid (Table S2).

## Results

### Plasmid profiling revealed that the majority of staphylococcal strains carry 20–30 kb plasmids with three major families

We screened 280 staphylococci, 247 of which had not previously been examined for plasmid content. Ninety percent of strains had plasmids, and 78.5% of these had one or more large plasmids >20 kb (Table S3). Of the 184 typable >20 kb plasmids in these newly screened strains, 75% were 20–30 kb (Table 1). Indeed, plasmids from 20 to 30 kb are extremely abundant among staphylococci, but they were only 13.7% of previously sequenced staphylococcal plasmids in RefSeq. Restriction types (RT) were assigned by RFLP analysis (see *Materials and Methods*), and three RTs (RT1, RT2, and RT3) encompassed 60.7% of typable 20–30 kb plasmids in strains not previously examined for plasmids (Table 2). These three RTs were also 42.5% of all large plasmids >20 kb typed and 49.0% of those from newly examined strains (Table 2). We assigned 106 distinct RTs, and 80% of those were unique to a single plasmid; thus, only those three RTs were widely common.

**Table 1:** Sizes of large typable staphylococcal plasmids

	Number of Plasmids (%)
Total typable >20 kb*^a^*	184
>30 kb	46 (25%)
20–30 kb	138 (75%)

a Large plasmids from newly examined strains (n = 247) typable by RFLP.

**Table 2:** RFLP patterns of staphylococcal plasmids reveal three prevalent families

Family	Restriction Type	Estimated Size (kb)*^a^*	Number of Plasmids*^b^*	20–30 kb*^d^*	>20 kb*^e^*
pMW2-like	RT1	18–21	27	19.6%	14.7%
pIB485-like	RT2	25–27	36*^c^*	26.1%	19.6%
pUSA300HOUMR-like	RT3	25–27	27	19.6%	14.7%
Total			90*^c^*	65.3%	49.0%

a Sizes estimated by comparisons of electrophoresed undigested DNA to size ladder (see *Materials and Methods*).

b Includes all typable plasmids >20 kb (n = 184) from newly examined strains.

c There was one additional pIB485-like plasmid in a strain previously examined for plasmids (for a total 37 of 214 typable >20 kb plasmids from all strains).

d The representation of that family among the typable 20–30 kb plasmids from newly examined strains (n = 138)

e The representation of that family among all typable >20 kb plasmids from newly examined strains (n = 184).

The RT1 family has the smallest plasmids of the three common groups, estimated by gels at 18–21 kb (Table 2), resembling previously sequenced pMW2 (Baba *et al.* 2002) and pSAS (Holden *et al.* 2004) of a U.S. MRSA strain and a U.K. MSSA strain, respectively. The RFLPs of 25–27 kb RT2 family resembled many previously unsequenced or partially sequenced plasmids, including 54 plasmids in California MRSA hospital strains isolated in the 1980s (Zuccarelli *et al.* 1990); plasmid pIB485 carrying the *sed* enterotoxin gene (Bayles and Iandolo 1989); and 8 pIB485-like *S. aureus* plasmids carrying *ser* and *sej* enterotoxin genes (Omoe *et al.* 2003). The RT3 plasmids also ranged from 25 to 27 kb (Table 2), but the RT3 restriction pattern resembled that of previously sequenced plasmid pUSA300HOUMR (NC_010063) (Highlander *et al.* 2007). Six pMW2-like RT1, 5 pIB485-like RT2, and 3 pUSA300HOUMR-like RT3 plasmids from diverse epidemiological and geographical backgrounds were chosen for sequencing, along with 86 plasmids with less common or unique RTs.

### Analysis of the sequenced staphylococcal plasmid genomes

Prior to submission of the sequences from this project, only 29 of the 102 complete staphylococcal plasmid sequences in RefSeq were larger than 20 kb (28%) (Table 3). The 93 new complete staphylococcal plasmid sequences (Table 3 and Table S2) increased the number of large >30 kb plasmids from 15 to 42, the number of 20–30 kb plasmids from 14 to 46, and the number of small <20 kb plasmids from 73 to 107. Thus, 45% of staphylococcal complete plasmid sequences are now >20 kb, which more closely represents the plasmid content observed in the 247 newly examined staphylococcal strains (Table S3). For the partial plasmid sequences that were deposited, 16 are plasmids with one small gap (∼20 bp), and the other 41 are large plasmid or phage contigs (1.4–56.7 kb) (Table S2). As expected (Malachowa and Deleo 2010; Novick 2003), the plasmids carry multiple antibiotic resistance genes and virulence genes, such as enterotoxins and exfoliative toxins (Table S2), including three new enterotoxins. Plasmid maintenance and transmissibility genes and transposons were also identified (Table S2).

**Table 3:** Complete staphylococcal plasmid genome sequences

	<20 kb	20–30 kb	>30 kb	Total
RefSeq	73	14	15	102
This project	34	32	27	93
Total	107	46	42	195

A RepA_N-type replication initiation gene (Weaver *et al.* 2009) occurs in 90% (54) of the 60 completely sequenced staphylococcal plasmids >10 kb [excepting the phage GQ900400, p5753cA GQ900435 from *E. faecium* and potential integrative conjugative element (ICE) GQ900429, Table S2]. The remaining 6, corresponding to pMW2-like RT1 plasmids, have only remnants of a RepA_N-type gene but contain a member of the Rep_3 superfamily first identified in the *S. epidermidis* plasmid pSK639 (Apisiridej *et al.* 1997; Firth and Skurray 2006). As further evidence of replicon fusions, 11 of 54 plasmids carrying a RepA_N-type gene also have a pSK639-like *rep* gene or a remnant thereof (Table S2). All 60 plasmids >10 kb encode some partitioning function: 78% have a homolog of the pSK1 *par* locus (Simpson *et al.* 2003), 12% have a type Ib partitioning system, and 10% have a type II system (Table S2).

#### Conjugation and mobilization loci:

Among plasmids large enough to encode conjugation loci (>12 kb) (Berg *et al.* 1998; Caryl and O'Neill 2009), only 6 of the 27 closed >30 kb plasmids have a clearly annotated conjugative transfer region, doubling the conjugation loci sequences available in RefSeq. An additional complete *tra* region was found in partial sequence SAP015B [GQ900500] (Table S2). Only 17 plasmids from this project (Table 4) have predicted *mob* loci (13 closed and 4 partial sequences), with only 3 from *S. aureus* (Table S2). An additional 31 plasmids (Table 4 and Table S2) encode a potential relaxase, *tra* or *pre*, which could function in mobilization (Francia *et al.* 2004; Garcillan-Barcia *et al.* 2009; Smith and Thomas 2004; Varella Coelho *et al.* 2009), for a total of 48 plasmids with putative mobilization genes.

**Table 4:** Predicted transfer loci in new staphylococcal plasmid sequences

	Total Sequences	*tra* Region	*Mob*	Relaxase (*pre* or *tra*)
Complete	93	6	13	22
Partial	57	1	4	9
Total	150	7	17	31

Transfer loci as predicted by RAST and/or P-RAST (http://rast.nmpdr.org) (Aziz *et al.* 2008).

#### *sin* and *res* plasmid maintenance recombinases:

Palindromic sequences in the recognition (res) sites, used by resolvases to separate plasmid multimers and thereby promote segregational stability, are also hotspots for insertion of Tn*552* and other transposons (Derbise *et al.* 1995; Lebard *et al.* 2008; Paulsen *et al.* 1994; Rowland and Dyke 1989, 1990). Such res sites are adjacent to plasmid resolvase *sin* genes in nonconjugative multiresistance plasmids and to *res* in the pSK41-like conjugative plasmids (Berg *et al.* 1998; Lebard *et al.* 2008; Rowland *et al.* 2002). Insertion of Tn*552*-, Tn*4002*-, or Tn*5404*-like transposons at these recombination hotspots produces a region of DNA flanked by inverted repeats (*resL* and *resR*) that can be inverted by the Bin recombinase (Derbise *et al.* 1995; Rowland and Dyke 1989, 1990; Rowland *et al.* 2002). This continual flipping process results in a region of sequence heterogeneity, complicating plasmid sequence assembly. The common occurrence of transposable elements such as Tn*552* and IS257 and recombinases Bin, Sin, and Res (Table 5 and Table S2) likely explains several of the 57 partial and gapped plasmid sequences we obtained.

**Table 5:** Recombinases and Tn*552* in staphylococcal plasmids

	Total	*res*	*sin*	*bin*	Tn*552*	ΔTn*552**^a^*	Tn*552*Δ*^b^*
RefSeq	102	7	16	20	14	4	0
This project							
Complete	93	7	44	40	10	23	2
Partial	57	1	32	28	7	12	3
Total	150	8	76	68	17	35	5

a ΔTn*552* includes *bin*, *blaI*, *blaR1*, and *blaZ* (Figure 1).

b Tn*552*Δ includes transposase genes and *bin* (Figure 1).

Forty-four complete staphylococcal plasmid sequences and 32 partial sequences carry the *sin* gene (Table 5). The *sin* gene occurs in 47% (44 of 93, Table 5) of staphylococcal plasmids completely sequenced in this project. *sin*, almost exclusively found on plasmids (where it is sometimes annotated as *bin3*), is not associated with a transposable element (Paulsen *et al.* 1994; Rowland and Dyke 1989; Rowland *et al.* 2002). A resolvase/invertase site-specific recombinase family member, *sin* most resembles Gram-positive resolvases like Res from *Enterococcus faecalis* plasmid pAMβ1 (Paulsen *et al.* 1994; Swinfield *et al.* 1991), which reduce multimers to ensure plasmid inheritance. *sin* may play this role for staphylococcal plasmids (Paulsen *et al.* 1994; Rowland *et al.* 2002). As expected (Berg *et al.* 1998; Lebard *et al.* 2008; Rowland *et al.* 2002), *sin* occurred on plasmids lacking *tra* genes, and *res* was found on the pSK41-like plasmids encoding conjugation genes, with only one exception: *res* also occurred on the mobilizable plasmid SAP016A from a *S. epidermidis* strain (Table S2). The same 75 bp internal deletion is in *sin* in 5 of the pMW2-like plasmids, and 8 newly sequenced *S. aureus* plasmids (4 complete sequences and 4 partial sequences) have truncated *sin* genes. Of the 60 complete staphylococcal plasmids >10 kb (Table S2) likely to require a resolvase, 51 or 85% have *sin* or *res*. Only 16 RefSeq plasmid sequences carry *sin* and only 7 have *res* (Table 5); in total, 74 of 195 (37.9%) complete staphylococcal plasmid sequences have a *sin* or *res* recombinase gene, presumably to enable stable plasmid inheritance.

#### Prevalence of bin recombinase and Tn552:

Besides *sin*, the single most common gene in the staphylococcal plasmids is *bin*, mostly found with the β-lactamase genes *blaZ*, *blaR1*, and *blaI* of the replicative transposon Tn*552* as the transposon’s resolvase. Of the plasmids >20 kb in size that we sequenced, 68 (68.7%) carry at least one *bin* (Table 5 and Table S2). Tn*552*, including *bin*, is also found on staphylococcal chromosomes (Rowland and Dyke 1989; Rowland and Dyke 1990). Full or truncated Tn*552* occurs on 53 complete staphylococcal plasmids (18 in RefSeq and 35 from this project, Table 5), isolated from strains found in Australia, the United Kingdom, the United States, and Columbia from the 1940s to the 2000s (Table S2), demonstrating intercontinental plasmid-mediated spread of antibiotic resistances and their persistence over six decades. We found the full Tn*552* located on 10 complete and 7 partial staphylococcal plasmids, and we found that 25 complete and 15 partial plasmids encode one of two truncated Tn*552* variants (Table 5).

Tn*552* encodes two transposition genes, p271 and p480, the resolvase *bin*, and the β-lactamase genes *blaI*, *blaR1*, and *blaZ*, and it generates 6 bp direct repeats of target DNA flanking its ∼120 bp inverted repeats (Rowland and Dyke 1989, 1990). The full or truncated variants of Tn*552* (Figure 1) occur in 38% (57 of 150) of the new complete and partial staphylococcal plasmid sequences but in only 18% of those in RefSeq (Table 5). Tn*552* insertion adjacent to transposons, within other transposons, or near res sites can create DNA segments that can then be inverted or deleted by Bin (Rowland and Dyke 1989, 1990). The prototypical ΔTn*552* in pI258 likely arose by Bin-mediated deletion (Rowland and Dyke 1990). In pI258, ΔTn*552* lacks transposition genes but has *bin* and all three β-lactamase–associated genes (Figure 1). Twenty-three complete and 12 partial newly sequenced staphylococcal plasmids, including pI258, have ΔTn*552*; only four examples of ΔTn*552* are in RefSeq. We first observed here the converse truncated version of Tn*552*, Tn*552*Δ, which has the transposase genes and *bin* but lacks the *bla* genes (Figure 1). Tn*552*Δ was only observed in 2 complete and 3 partial staphylococcal plasmids (Table 5).

**Figure 1:**
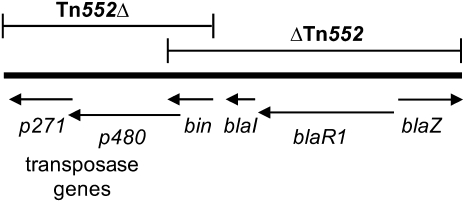
Dominant variants of Tn*552* found on *Staphylococcus* plasmids. The full-length Tn*552* is characterized by two transposase genes, *p271* and *p480*, a *bin* recombinase, and three β-lactamase–associated genes, *blaI*, *blaR1*, and *blaZ*. Tn*552*Δ is missing the *bla* genes, and ΔTn*552* is missing the transposase genes.

### Three major *Staphylococcus* plasmid families revealed by plasmid profiling were confirmed and extended by sequencing

#### pIB485-like enterotoxin plasmids are found worldwide:

**Figure 2:**
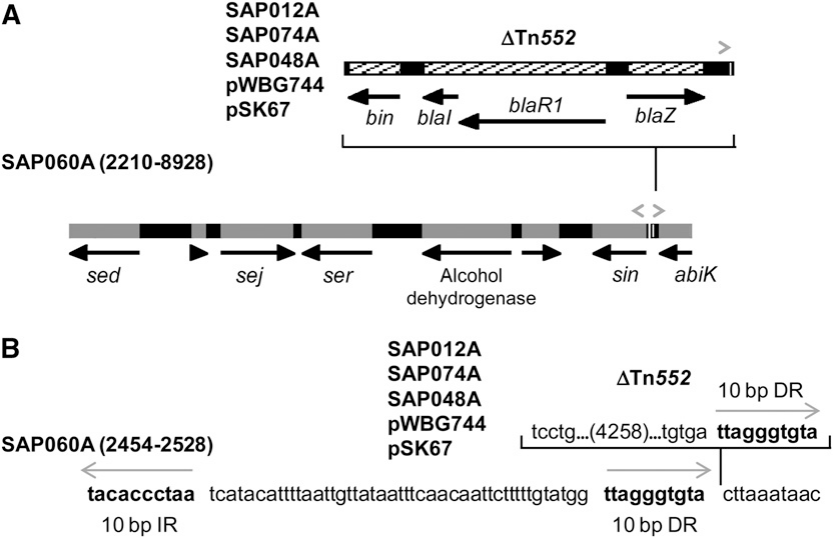
pIB485-like enterotoxin plasmids with or without ΔTn*552*. (A) Diagram of pIB485-like plasmid SAP060A (open reading frames in gray), positions 2210–8928, showing the insert position of the 4278 bp ΔTn*552* (hatched) in the *sin* res site (Rowland *et al.* 2002) in the other pIB485-like plasmids (Table 6). The ΔTn*552* (hatched) insert has flanking 10 bp direct repeats (right-facing gray arrowheads) of res site DNA, likely resulting from transposition. An inverted repeat of that same 10 bp insertion site is also present upstream from *sin*, as part of the predicted res site (left-facing gray arrowhead). The black arrows show predicted open reading frames. (B) The sequence details of the ΔTn*552* inserted in the *sin* res site. The sequence without the insertion is SAP060A positions 2454–2528. The other pIB485-like plasmid sequences contain ΔTn*552*, and the nucleotides shown are identical in all. The gray arrows show the 10 bp direct and inverted repeats.

At 26% of 20–30 kb newly profiled plasmids, the pIB485-like RT2 plasmids are the most prevalent of the three major RTs identified by RFLP profiling (Table 2). No pIB485-like plasmid had been completely sequenced before this project, but 63 plasmids with a similar RFLP had been reported (see above) (Bayles and Iandolo 1989; Omoe *et al.* 2003; Zuccarelli *et al.* 1990), including the SED enterotoxin–containing plasmid pIB485 (Bayles and Iandolo 1989). The five strains whose plasmids we sequenced were isolated between 1949 and 2001 (Table 6) in the United States (2), the United Kingdom (1), and Australia (2), and they have ΔTn*552* (Figure 1) similar to that of pI258. Over 99% identical (Table 6), these five plasmids clearly demonstrate geographic spread and stability in terms of gene sequence and organization over five decades. A sixth otherwise identical plasmid, SAP060A, from a pre-1960 US isolate, lacks ΔTn*552* (Figure 2A) but is otherwise 99.97% identical to SAP012A (Table 6), a pIB485-like plasmid isolated from a 1995 US MRSA strain. In the other five pIB485-like plasmids, a 10 bp direct repeat of plasmid DNA flanks the ΔTn*552* segment (Figure 2B), suggesting it was inserted by transposition; this same 10 bp also occurs as an inverted repeat in the predicted *sin* res site (Rowland *et al.* 2002). Thus, SAP060A has two copies of the 10 bp sequence in an inverted repeat, and the 5 other pIB485-like plasmids have three copies of the 10 bp sequence, two in direct repeat flanking ΔTn*552* and the third on the opposite strand (Figure 2B).

**Table 6:** Completely sequenced pIB485-like *S. aureus* plasmids

Plasmid	Percentage Identity to SAP012A*^a^*	Strain Source	Year Isolated	Location
SAP012A	—	Human clinical	1995	Georgia, United States
SAP048A	99.97	Human clinical	2006	Nebraska, United States
SAP074A	99.90	CA infection	1999	Oxford, United Kingdom
pWBG744	99.96	Screening	2001	Western Australia
pSK67	99.30	Human clinical	1949	Melbourne, Australia
SAP060A	84.29 (99.97)*^b^*	Not from infection	Pre-1960	United States

CA, community-acquired.

a Mismatches and gaps were determined by ClustalW alignment. Gaps were considered mismatches in calculating percentage identity.

b Percentage identity to SAP012A without ΔTn*552*.

In addition to ΔTn*552*, all pIB485-like plasmids, including SAP060A, carry the cluster of *Staphylococcus* enterotoxin genes *sed*, *sej*, and *ser* previously reported in this plasmid family (Bayles and Iandolo 1989; Omoe *et al.* 2003) that have contributed to several outbreaks of *S. aureus* food-borne illness (Omoe *et al.* 2003; Ono *et al.* 2008; Plata *et al.* 2009). The SAP012A and SAP060A sequences have a frameshift in *sed* due to a missing T in a run of eight Ts. This may be an occurrence of a common sequencing error (Li and California 2006), as only those two sequences have the *sed* frameshift; the question can be resolved by testing for the presence of the relevant gene transcript or product. The pIB485-like plasmids also carry cadmium resistance genes *cadX* and *cadD*. Energy-dependent Cd(II) efflux was first described by Tynecka (Tynecka *et al.* 1981a, 1981b) with pII147 and later in several staphylococcal plasmids, including pI258 (Crupper *et al.* 1999; Massidda *et al.* 2006; Nies 1992; Nucifora *et al.* 1989).

#### pMW2-like plasmids occur in human and animal S. aureus strains:

**Figure 3:**
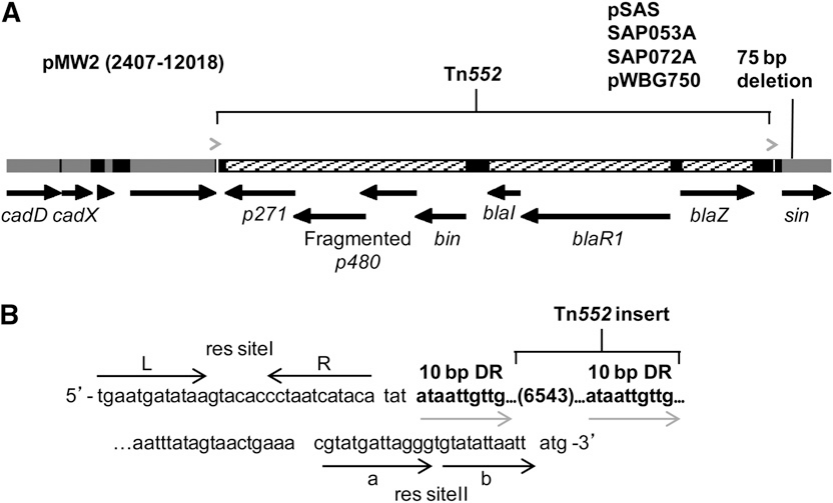
pMW2-like plasmids with full-length Tn*552*. (A) Diagram of the pMW2 sequence positions 2407–12018, including the complete Tn*552* with flanking 10 bp direct repeats (gray arrowheads). All pMW2-like plasmids (Table 7) have a frameshift that truncates the *p480* transposase and generates a second putative ORF. pMW2 and the four plasmids listed have a 75 bp deletion in *sin*; the four remaining pMW2-like plasmids have the full *sin* gene. (B) The pMW2 sequence that flanks Tn*552*, positions 4831–11469. Tn*552* (6553 bp, including the 10 bp DR) is inserted into the putative *sin* res site; res siteI and res siteII predicted for p21 (Rowland *et al.* 2002) are labeled (black arrows). This sequence is identical in all pMW2-like plasmids. The gray arrows show the 10 bp direct repeats.

Like the pIB485-like plasmids, the pMW2-like RT1 plasmids are common (Table 2) with a wide geographical distribution, composing ∼20% (27 of 138) of the 20–30 kb plasmids and coming from US, UK, and Australian *S. aureus* strains between 1995 and 2004 (Table 7). Only three pMW2-like plasmids (pMW2, pSAS, and p21) were available in RefSeq when we sequenced the six described here. All pMW2-like plasmids are 99% identical (Table 7), differing mainly by a 75 bp deletion in *sin* only in pMW2, pSAS, SAP053A, SAP072A, and pWBG750 (Figure 3A). SAP072A, from the most recently isolated *S. aureus* strain (2004 UK), was from an animal isolate (Table 7); thus, these plasmids are not limited to human strains. SAP072A is 99.99% identical to pMW2, a plasmid from a 1998 US human CA-MRSA clinical isolate (Baba *et al.* 2002).

**Table 7:** Completely sequenced pMW2-like *S. aureus* plasmids

Plasmid	Percentage Identity to pMW2*^a^*	Strain Source	Year	Location	Reference*^b^*
pMW2	—	Human clinical	1998	North Dakota, United States	Baba *et al.* 2002
pSAS	99.93	Human clinical	1998	United Kingdom	Holden *et al.* 2004
p21	98.95	Clinical	unknown	unknown	Unpublished
SAP053A	99.87	Human clinical	2007	Nebraska, United States	This work
SAP072A	99.99	Animal clinical	2004	United Kingdom	This work
SAP073A	99.58	CA infection	1999	Oxford, United Kingdom	This work
pWBG750	99.96	CA infection	1995	Western Australia	This work
pWBG757	99.54	Screening	1995	Western Australia	This work
pWBG763	99.58	Screening	1995	Western Australia	This work

CA, community-acquired.

a Mismatches and gaps were determined by ClustalW alignment. Gaps were considered mismatches in calculating percentage identity.

b See Table S2 for accession numbers of newly sequenced plasmids. Previously sequenced plasmids are pMW2, NC_005011; pSAS, NC_005951; and p21, NC_002517.

The pMW2-like RT1 plasmids are the smallest (20.7 kb) of the prevalent RT groups (Table 7). They carry putative bacteriocin and bacteriocin-immunity genes and several short ORFs annotated as *pre*, indicating possible mobilizability (Francia *et al.* 2004; Garcillan-Barcia *et al.* 2009; Smith and Thomas 2004; Varella Coelho *et al.* 2009). As with the pIB485-like plasmids, the pMW2-like plasmids carry cadmium resistance (*cadD*, *cadX*) and have a full Tn*552* in the *sin* res site predicted for p21 (Rowland *et al.* 2002) (Figure 3, A and B). All pMW2-like plasmids contain a frameshift mutation in the Tn*552* transposase *p480*, fragmenting it into two separate, overlapping predicted open reading frames (Figure 3A), both of which encode putative transposases, unlike the prototypical Tn*552* of pI9789 where *p480* is a single gene (Rowland and Dyke 1989, 1990). Like the ΔTn*552* in the pIB485-like plasmids (Figure 2, A and B), the full Tn*552* in the RT1 plasmids is flanked by 10 bp direct repeats of plasmid DNA (Figure 3B). Although no pMW2-like plasmids lacking Tn*552* have been reported, such a plasmid (14.1 kb) would be much smaller than the >20 kb plasmids we focused on because the Tn*552* insert is 6553 bp.

#### pUSA300HOUMR-like multiresistance plasmids are common in the United States:

The pUSA300HOUMR-like RT3 plasmids are the third common plasmid group identified by RFLP analysis and, like the pMW2-like plasmids, comprised ∼20% of the 20–30 kb plasmids we typed (Table 2). Unlike the geographically diverse pIB485-like and pMW2-like plasmids, they were found only in US *S. aureus* isolates (Table 8). However, like the pMW2-like plasmids, the pUSA300HOUMR-like RT3 plasmids occurred in both animal and human strains. The three plasmids identified as RT3 by RFLP analysis are 99.8–99.9% identical at the sequence level (Table 8) to plasmid pUSA300HOUMR from a human clinical MRSA isolated in Houston, TX (Highlander *et al.* 2007). Four other pUSA300HOUMR-like plasmids were identified by sequencing to have >98% identity with pUSA300HOUMR, apart from insertions and deletions (Table 8).

**Table 8:** Completely sequenced pUSA300HOUMR-like *S. aureus* plasmids

Plasmid	Percentage Identity to pUSA300HOUMR*^a^*	Strain Source	Year	Location	Reference*^c^*
pUSA300HOUMR	—	Human clinical	2002–4	Texas, United States	Highlander *et al.* 2007
SAP015A	99.83	Human clinical	2002	California, United States	This work
SAP046A	99.89	Canine abscess	2005	Georgia, United States	This work
SAP050A	99.89	Human clinical	2007	Nebraska, United States	This work
SAP027A	89.58 (98.04)*^b^*	Human clinical	2006	Nebraska, United States	This work
SAP049A	92.31 (99.88)*^b^*	Human clinical	2007	Nebraska, United States	This work
SAP051A	85.06 (99.89)*^b^*	Human clinical	2007	Nebraska, United States	This work
SAP052A	83.28 (99.85)*^b^*	Human clinical	2007	Nebraska, United States	This work

a Mismatches and gaps were determined by ClustalW alignment. Gaps were considered mismatches in calculating percentage identity.

b Percentage identity to pUSA300HOUMR without largest gap (insertion or deletion) (see Figure 4).

c pUSA300HOUMR, NC_010063; other accession numbers are in Table S2.

All pUSA300HOUMR-like plasmids carry a ΔTn*552* (*bin*, *blaI*, *blaR1*, *blaZ*) (Figure 4) similar to pI258 and the pIB485-like RT2 plasmids, but it is not flanked by 10 bp repeats or located within the truncated *sin* res site. Lacking the Tn*552* transposases, the pUSA300HOUMR-like plasmids have a transposase similar to that carried by IS257 and IS431mec, three copies of which are found in these plasmids. A deletion event like that described for pI258 (Rowland and Dyke 1990) may have removed the Tn*552* transposases adjacent to *bin*.

**Figure 4:**
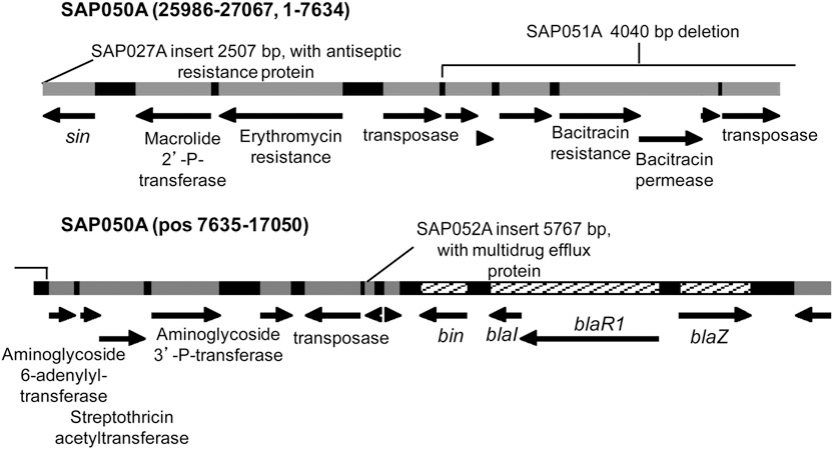
Multiresistant pUSA300HOUMR-like plasmids with a ΔTn*552* that lacks flanking direct repeats. Diagram of the pUSA300HOUMR-like plasmid SAP050A, positions 25986 through the end of the sequence (27067) and positions 1 to 17050, showing insertions and deletions for the other pUSA300HOUMR-like plasmids (Table 8). The ΔTn*552* genes are hatched; all other predicted genes are gray. SAP027A has a 923 bp region of high mismatch (to positions 25058–25980) and a 2507 bp insertion just prior to the illustrated sequence that includes a predicted antiseptic resistance protein. SAP052A has a 5767 bp insertion after position 11570 that includes a predicted multidrug efflux protein. SAP051A does not encode bacitracin resistance genes (deleted positions 3654–7693). The SAP049A 2047 bp deletion is not shown (positions 19771–21817) and includes three hypothetical genes upstream of *cadX*.

Besides the β-lactamase and cadmium resistances carried by the pIB485-like and pMW2-like plasmids, the pUSA300HOUMR-like plasmids also encode macrolide and aminoglycoside resistances, and all but SAP015A carry bacitracin resistance (Figure 4). SAP027A has an insertion encoding antiseptic resistance, and SAP053A has an additional predicted multidrug efflux protein (Figure 4). The four non-RT3 pUSA300HOUMR-like plasmids identified by sequencing, because insertions and/or deletions gave them different RFLP profiles, show how easily these plasmids may gain even more resistance genes. Eight additional plasmids belonging to this family were recently sequenced from clinical *S. aureus* USA300 isolates from multiple locations in the United States (Kennedy *et al.* 2010).

## Discussion

The prevalence of 20–30 kb plasmids, almost half of which belong to only three restriction types by RFLP analysis, in staphylococci isolated from sources very distant in time and space suggests that these nonconjugative plasmids are surprisingly widespread for non–self-mobile plasmids. Plasmids in this size range can potentially be transferred by transducing phages (Lindsay and Holden 2006; Malachowa and Deleo 2010; Smillie *et al.* 2010); most phage genomes identified in staphylococci are >40 kb, and transduction is thought to be restricted by phage genome size (Smillie *et al.* 2010). More of these 20–30 kb plasmids may be mobilizable than is apparent with current genome data if they contain *mob* genes not yet identified as such. However, the scarcity of conjugative plasmids (only 12 in total) implies that mobilization is rare and that staphylococcal plasmid transfer occurs mainly by transduction (Lindsay and Holden 2004; Lindsay 2010). The now larger dataset makes the mechanism of intercellular movement of these strongly peripatetic 20–30 kb plasmids ripe for examination.

Serine recombinases important for many mobile elements are prevalent in staphylococci (Rowland *et al.* 2002), playing roles in stable plasmid inheritance and transposon movement. Sin recombinase may have a role in plasmid multimer resolution (Rowland *et al.* 2002) in 47% of staphylococcal plasmids completely sequenced in this project and in 31% of all complete sequences, including those in RefSeq. Other serine recombinases are associated with the movement of antibiotic resistances, such as transposition of vancomycin resistance genes on Tn*1546* (Katayama *et al.* 2000) and the β-lactamase genes carried on Tn*552* whose movement is associated with *bin*. In addition to being the Tn*552* resolvase, *bin* is commonly found on large staphylococcal plasmids and may play a role in deletions and rearrangements (Murphy and Novick 1980; Rowland and Dyke 1989, 1990).

The β-lactamase–encoding transposon Tn*552* in plasmids of staphylococci isolated between the 1940s and the 2000s from distant locations shows the persistence of antibiotic resistance genes over time and geography. All three major families we observed carried β-lactamase genes associated with partial or full Tn*552* and with cadmium resistance genes. Virtually identical pIB485-like plasmids with (or without) a ΔTn*552* and carrying three enterotoxin genes (Figure 2A) occurred in strains from distant locations isolated 50 years apart. Our work markedly expands and enriches the evidence that plasmids have retained and gained gene content as they spread across the globe among epidemiologically and geographically diverse *S. aureus* strains.

Sequence alignments revealed four pUSA300HOUMR-like plasmids not identified as such by RFLP analysis, emphasizing the spread of this family among US *S. aureus* strains and showing they are even more abundant than detectable by plasmid profiling (Table 2). The pUSA300HOUMR-like plasmids all carry multiple antimicrobial resistances, and two of them, SAP027A and SAP052A, have insertions with additional resistance genes (Figure 4). These plasmids are a snapshot of how easily resistance genes are gained and spread among pathogenic staphylococci. The pUSA300HOUMR-like group of plasmids was found only in *S. aureus* strains isolated in the United States, unlike the other two major families, but it is likely that these plasmids will spread worldwide as have the pMW2-like and pIB485-like plasmids.

The three major families identified show that these plasmids are persistent and widespread on a global scale. The sequenced pMW2-like (Table 7) and pIB485-like (Table 6) plasmids were from strains isolated on three continents, and the latter group's strains were isolated decades apart (Table 6). The pMW2-like and pUSA300HOUMR-like plasmids came from both human and animal isolates, reemphasizing (Lindsay 2010) that properly assessing the spread of *S. aureus* strains and their mobile elements requires study of both animal and human strains to determine whether human strains are infecting animals or whether strains are simply sharing mobile elements.

In summary, we aimed to increase the number of large staphylococcal plasmid sequences to better assess the global and temporal diversity and spread of these mobile elements. We have tripled the number of large plasmid sequences available and identified three major plasmid families and the most common genes found on large plasmids, opening several key areas for future investigation. The phylogeny of the plasmids should be examined, but their varying sizes and the large number and variety of their transposable elements challenges classical cladistics approaches. These plasmids also carry several classes of genes providing clues to their ecology within the worldwide population of staphylococci. The abundance of arsenic, mercury, and cadmium resistances suggests that nonantibiotic environmental toxicants foster persistence of these multiresistant mobile elements. Arsenic is widely used in animal agriculture (Jackson and Bertsch 2001; Rutherford *et al.* 2003), approximately 30% of the US population is directly exposed to mercury via dental amalgam restorations (Richardson *et al.* 2011), and cadmium exposure can be occupational (Cespon-Romero and Yebra-Biurrun 2007; Wang *et al.* 2008) or from tobacco use (Butler Walker *et al.* 2006). There are several enterotoxins, exfoliative toxins, and *pls* (antiadhesion to nasal epithelial cells) (Savolainen *et al.* 2001), as well as predicted bacteriocins/lantibiotic genes and a very common predicted *abiK* gene (Table S2) that may have antibacterial or antiviral effects, any or all of which might be involved in the equilibrium between being a benign commensal or life-threatening pathogen. The mechanism of spread of these plasmids, including the potential for mobilization, especially of those shared among animal and human strains, is particularly important to elucidate.

## Supplementary Material

Supporting Information
