# Supplementary material for: Major Families of Multiresistant Plasmids from Geographically and Epidemiologically Diverse Staphylococci
Source: G3 (Bethesda). 2011 Dec 1;1(7):581–91. doi: 10.1534/g3.111.000760 (PMC3276174; doi:10.1534/g3.111.000760)
Supplement: Supporting Information [file supp_1_7_581__index.html]

Supporting Information 

# Major Families of Multiresistant Plasmids from Geographically and Epidemiologically Diverse Staphylococci

## Supporting Information for Shearer *et al.*, 2011

**Files in this Data Supplement:**

- Supporting Information - Tables S1-S3 (PDF, 256 KB)
- Table S1 - Strain collections used in this work (PDF, 60 KB)
- Table S2 - Strain information and restriction types for *Staphylococcus* (and two *Enterococcus*) plasmid sequences obtained (PDF, 248 KB)
- Table S3 - Large plasmid content of newly screened *Staphylococcus* strains (PDF, 72 KB)
