# Supplementary material for: Major Families of Multiresistant Plasmids from Geographically and Epidemiologically Diverse Staphylococci
Source: G3 (Bethesda). 2011 Dec 1;1(7):581–91. doi: 10.1534/g3.111.000760 (PMC3276174; doi:10.1534/g3.111.000760)
Supplement: Supporting Information [file supp_1.7.581_000760SI.pdf]

**Table S1 Strain collections used in this work**

| Strain collection | Source | Description | Number screened |
|---|---|---|---|
| NARSA[a] | NARSA[a], US | Human clinical | 10 |
| CDC[b] | Centers for Disease Control and Prevention, US | Human clinical | 64[b] |
| Veterinary | University of Georgia, GA, US | Companion animal | 14 |
| Poultry | University of Georgia, GA, US | Poultry litter | 8 |
| Nebraska | University of Nebraska Medical Center, NE, US | Human clinical | 51 |
| Western Australia | Curtin University of Technology, Perth, Western Australia | Human clinical | 35 |
| United Kingdom | University of London, London, UK | Human and animal clinical, screenings and infections | 65 |
| Historic | University of Sydney, Sydney, AU | Human clinical | 29 |
| **Total** | | | **280** |

[a] Network on Antimicrobial Resistance in *Staphylococcus aureus* (NARSA) (http://www.narsa.net)
[b] There were four additional transconjugant strains with plasmids from human clinical strains in this collection (McDougal *et al*. 2008).

**Table S2 Strain information and restriction types for *Staphylococcus* (and two *Enterococcus)* plasmid sequences obtained.[a]**

| Accession | Plasmid | Size | Strain | Species | Plasmid Genotype[b] | RT | Sequence | Year | Location | Status | Other strain info[c] | Plasmid phenotype[d] | Identified by |
|---|---|---|---|---|---|---|---|---|---|---|---|---|---|
| GQ900386 | SAP020A | 64909 | CDC3 | *S. sp. (CNS)* | *bin*, *repA,* Type Ib partitioning | RT44 | complete | 1996 | GA, USA | human clinical isolate | OxS | | Jean Patel, Ainsley Nicholson, Brandi Limbago: Division of Healthcare Quality Promotion, Centers for Disease Control and Prevention, Atlanta, GA, USA |
| GQ900421 | SAP068A | 50500 | PM64 | *S. aureus* | *aacA-aphD, dfr, tcaA, tra, res*, Tn*552*, IS256 x2, *repA*, Type II partitioning | RT83 | complete | 1999 | SGH, London, UK | clinical, hospital-acquired infection | HA-MRSA | | Jodi Lindsay. Department of Cellular and Molecular Medicine, St. George's, University of London, London, UK |
| GQ900379 | SAP014A | 50429 | CDC58 | *S. aureus* | *tra, res, aacA-aphD*, IS256 x2, *tcaA, tet, pre, dfr,* ble, kan, *repA,* Type II partitioning | RT47 | complete | 2000 | MS, USA | human clinical isolate | HA-MRSA | | Jean Patel, Ainsley Nicholson, Brandi Limbago: Division of Healthcare Quality Promotion, Centers for Disease Control and Prevention, Atlanta, GA, USA |
| GQ900432 | SAP079A | 47322 | CDCGA672 | *S. aureus* | *tra, res, aacA-aphD*, IS256 x2, *tcaA,* kan, multidrug resistance, *repA*, Type II partitioning | RT112 | complete | | GA, USA | human clinical isolate | | | Jean Patel, Ainsley Nicholson, Brandi Limbago: Division of Healthcare Quality Promotion, Centers for Disease Control |

| | | | | | | | | | | | | | |
|---|---|---|---|---|---|---|---|---|---|---|---|---|---|
| | | | | | | | | | | | | | and Prevention, Atlanta, GA, USA |
| GQ900448 | pSK156 | 45052 | SK1271 | *S. aureus* | *cadXD,* antiseptic resistance, *sin,* ΔTn*552*, *se,* bacteriocin, *repA, par* | RT97 | complete | 1951 | Melbourne, Australia | human clinical isolate | | AsaPcQacB | Ron Skurray, Neville Firth, Slade Jensen: School of Biological Sciences, University of Sydney, Sydney, AU |
| GQ900434 | SAP082A | 44116 | CDCPANICU | *S. aureus* | *tra, res*, kan, *tcaA,* IS256 x2, *aacA-aphD,* multidrug efflux, *repA*, Type II partitioning | RT115 | complete | | PA, USA | human clinical isolate | | | Jean Patel, Ainsley Nicholson, Brandi Limbago: Division of Healthcare Quality Promotion, Centers for Disease Control and Prevention, Atlanta, GA, USA |
| GQ900433 | SAP080A | 43892 | CDCTN147 | *S. aureus* | *tra, res*, ble, kan, IS256 x2, *aacA-aphD, tcaA, dfr, repA*, Type II partitioning | RT113 | complete | | TN, USA | human clinical isolate | | | Jean Patel, Ainsley Nicholson, Brandi Limbago: Division of Healthcare Quality Promotion, Centers for Disease Control and Prevention, Atlanta, GA, USA |
| GQ900381 | SAP016A | 43807 | CDC8 | *S. epidermidis* | *aacA-aphD,* ble, kan, multidrug efflux, *mob, pre, res, tcaA,* ΔTn*552*, IS256 x2, *repA, rep, par* | RT51 | complete | 1999 | CA, USA | human clinical isolate | OxS | | Jean Patel, Ainsley Nicholson, Brandi Limbago: Division of Healthcare Quality Promotion, Centers for Disease Control and Prevention, Atlanta, GA, USA |

| | | | | | | | | | | | | |
|---|---|---|---|---|---|---|---|---|---|---|---|---|
| GQ900456 | SAP107A | 42623 | SK30 | *S. epidermidis* | *bin, cadXD*, multidrug efflux, asp/sec, *repA*, Type Ib partitioning | RT106 | complete | 1980 | Melbourne, Australia | human clinical isolate | | Ron Skurray, Neville Firth, Slade Jensen: School of Biological Sciences, University of Sydney, Sydney, AU |
| GQ900422 | SAP069A | 42198 | PM79 | *S. aureus* | *tra, res*, IS256 x2, *aacA-aphD,* kan, *tcaA,* multidrug efflux, *repA*, Type II partitioning | RT41 | complete | 1999 | SGH, London, UK | clinical, hospital-acquired infection | HA-MRSA | Jodi Lindsay. Department of Cellular and Molecular Medicine, St. George's, University of London, London, UK |
| GQ900415 | SAP057A | 39308 | 3049 | *S. aureus* | *cadXD, etb*, bacteriocin, exo, *pls, repA*, Type Ib partitioning | RT26 | complete | 1999 | Oxford, UK | not from infection | MSSA | Jodi Lindsay. Department of Cellular and Molecular Medicine, St. George's, University of London, London, UK |
| GQ900454 | SAP106A | 38556 | SK939 | *S. epidermidis* | *ars, mob, sin*, ΔTn552, bacteriocin/lantibiotic, *repA, rep*, Type Ib partitioning | RT104 | complete | 1946 | Melbourne, Australia | human clinical isolate | | Ron Skurray, Neville Firth, Slade Jensen: School of Biological Sciences, University of Sydney, Sydney, AU |
| GQ900389 | pWBG745 | 38204 | WB43S | *S. aureus* | *tra* (incomplete), *repA*, Type Ib partitioning | RT11 | complete | 2001 | Remote Western Australia | screening | ST73-MRSA-IVa (2B) | Frances O'Brien. School of Biomedical Sciences, Curtin University of Technology, Perth, Western Australia |

| | | | | | | | | | | | | |
|---|---|---|---|---|---|---|---|---|---|---|---|---|
| GQ900391 | pWBG749 | 38087 | WBG8381 | *S. aureus* | *tra* (incomplete), *repA*, Type Ib partitioning | RT10 | complete | 1995 | Remote Western Australia | screening | ST5-MRSA-IVa (2B) | Frances O'Brien. School of Biomedical Sciences, Curtin University of Technology, Perth, Western Australia |
| GQ900400 | pWBG758 | 38045 | Y74T | *S. aureus* | only phage genes | RT16 | complete | 1996 | Remote Western Australia | screening | ST761-MSSA | Frances O'Brien. School of Biomedical Sciences, Curtin University of Technology, Perth, Western Australia |
| GQ900402 | SAP045A | 37564 | CDC9 | *S. epidermidis* | *bla,* bacitracin, multidrug resistance, *sin, repA, par* | RT62 | complete | 2000 | FL, USA | human clinical isolate | OxS | Jean Patel, Ainsley Nicholson, Brandi Limbago: Division of Healthcare Quality Promotion, Centers for Disease Control and Prevention, Atlanta, GA, USA |
| GQ900427 | SAP076A | 35114 | PM86 | *S. aureus* | *ars, bin, cadCA, pre, sin*, metal transporting ATPase, *repA, rep, par* | RT30 | complete | 1999 | SGH, London, UK | not from infection | HA-MRSA | Jodi Lindsay. Department of Cellular and Molecular Medicine, St. George's, University of London, London, UK |
| GQ900428 | SAP077A | 35510 | 879R4RF | *S. aureus* | *ars, bin, cadCA, pre, sin*, metal transporting ATPase, biofilm-associated protein, *repA, rep, par* | RT31 | complete | 1982 | USA | not from infection | MSSA | Jodi Lindsay. Department of Cellular and Molecular Medicine, St. George's, University of |

| | | | | | | | | | | | | |
|---|---|---|---|---|---|---|---|---|---|---|---|---|
| | | | | | | | | | | | | London, London, UK |
| GQ900430 | SAP078A | 35508 | A78R1 | *S. aureus* | *ars, bin, cadCA, pre, sin*, metal transporting ATPase, *repA, rep, par* | RT31 | complete | 2007 | UK | not from infection | MRSA | Jodi Lindsay. Department of Cellular and Molecular Medicine, St. George's, University of London, London, UK |
| GQ900390 | pWBG746 | 33702 | WB43S | *S. aureus* | *cadXD, sin, se*, bacteriocin, exo, *repA, par* | RT12 | complete | 2001 | Remote Western Australia | screening | ST73-MRSA-IVa (2B) | Frances O'Brien. School of Biomedical Sciences, Curtin University of Technology, Perth, Western Australia |
| GQ900399 | pWBG747 | 33701 | K153N | *S. aureus* | *cadXD, se*, bacteriocin, exo, *sin, repA, par* | RT12 | complete | 1995 | Remote Western Australia | screening | ST73-MSSA | Frances O'Brien. School of Biomedical Sciences, Curtin University of Technology, Perth, Western Australia |
| GQ900387 | pCM05 | 33660 | CM05 | *S. aureus* | *ars, cadCA, cadXD, mer, sin* x2, Tn*552*, *bin, repA, par* | RT84 | complete | 2005 | Medellin, Colombia | human clinical pneumonia isolate, sputum | ST5-MRSA-mec I | Alexander Mankin: Center for Pharmaceutical Biotechnology, University of Illinois, Chicago, IL, USA |
| GQ900382 | SAP017A | 32650 | VET A0-49420c | *S. aureus* | *aacA-aphD, dfr, cadXD*, Tn*552*Δ, *blaZ*, IS256 x3, *repA, par* | RT63 | complete | 2000 | Hendersonville Co, NC, USA | equine abscess isolate | USA500 t064 ST250-mec IVa | Susan Sanchez: Department of Infectious Diseases, College of Veterinary Medicine, University |

| | | | | | | | | | | | | |
|---|---|---|---|---|---|---|---|---|---|---|---|---|
| | | | | | | | | | | | | of Georgia, Athens, GA, USA |
| GQ900383 | SAP018A | 32487 | CDC25 | *S. sp. (CNS)* | *aacA-aphD, dfr, mob*, putative nickase, ΔTn*552*, *sin,* IS256 x2, *repA, rep, par* | RT65 | complete | 2001 | AR, USA | human clinical isolate | OxR | Jean Patel, Ainsley Nicholson, Brandi Limbago: Division of Healthcare Quality Promotion, Centers for Disease Control and Prevention, Atlanta, GA, USA |
| GQ900412 | SAP052A | 32445 | NE 3885 | *S. aureus* | *cadXD, sin*, ΔTn*552*, *aph,* sta, *aad*, bacitracin resistance, mac, ery, multidrug efflux, *repA, par* | RT6 (RT3-like) | complete | 2007 | NE, USA | human clinical isolate, wound | | Kenneth Bayles: Department of Pathology and Microbiology, University of Nebraska Medical Center, Omaha, NE, USA |
| GQ900419 | SAP064A | 30857 | EMRSA-3 | *S. aureus* | *cadCA, cadXD, mer,* antiseptic resistance, ΔTn*552, sin, repA, par* | RT35 | complete | 1987 | UK | clinical, hospital-acquired infection | HA-MRSA | Jodi Lindsay. Department of Cellular and Molecular Medicine, St. George's, University of London, London, UK |
| GQ900395 | pWBG753 | 30047 | WBG7583 | *S. aureus* | *cadCA, tet*, Tn*552*, *sin, pre, repA, par* | RT14 | complete | 1995 | Remote Western Australia | screening | ST8-MRSA-IVa (2B) | Frances O'Brien. School of Biomedical Sciences, Curtin University of Technology, Perth, Western Australia |

| | | | | | | | | | | | | | |
|---|---|---|---|---|---|---|---|---|---|---|---|---|---|
| GQ900388 | SAP027A | 29646 | NE 3828 | *S. aureus* | *cadXD, sin*, ΔTn*552*, *aph,* sta, *aad,* bacitracin resistance, mac, ery, antiseptic resistance, *repA, par* | RT7 (RT3-like) | complete | 2006 | NE, USA | human clinical isolate, wound | | | Kenneth Bayles: Department of Pathology and Microbiology, University of Nebraska Medical Center, Omaha, NE, USA |
| GQ900378 | pI258 | 29254 | NRS128 | *S. aureus* | *ars, cadCA, mer, sin*, ΔTn*552*, mac-linc-str-ery, *repA, par* | RT66 | complete | pre-1963 | | | | | |
| GQ900405 | SAP047A | 28974 | NE 3008 | *S. aureus* | *cadXD, sin*, ΔTn*552*, *pre, ser, sej, ses, set, repA, rep, par* | RT4 | complete | 2005 | NE, USA | human clinical isolate, synovial fluid | | | Kenneth Bayles: Department of Pathology and Microbiology, University of Nebraska Medical Center, Omaha, NE, USA |
| GQ900401 | pWBG759 | 28384 | C57S | *S. aureus* | *cadXD, sin*, ΔTn*552*, *sdrE, repA, par* | RT19 | complete | 1995 | Remote Western Australia | screening | ST20-MSSA | | Frances O'Brien. School of Biomedical Sciences, Curtin University of Technology, Perth, Western Australia |
| GQ900447 | pSK67 | 27439 | SK1027 | *S. aureus* | *cadXD, ser, sej, sed, sin*, ΔTn*552*, *repA, par* | RT2 | complete | 1949 | Melbourne, Australia | human clinical isolate | | CdPc | Ron Skurray, Neville Firth, Slade Jensen: School of Biological Sciences, University of Sydney, Sydney, AU |
| GQ900385 | SAP019A | 27435 | NRS104 | *S. aureus* | *ars, cadCA, bin, sin, pre*, bacteriocin, metal | RT73 | complete | 1935 | USA | | | | |

| | | | | | | | | | | | | |
|---|---|---|---|---|---|---|---|---|---|---|---|---|
| | | | | | transporting ATPase, *repA, rep, par* | | | | | | | |
| GQ900426 | SAP074A | 27268 | 433 | *S. aureus* | *abiK, cadXD, ser, sej, sed, sin*, ΔTn*552, repA, par* | RT2 | complete | 1999 | Oxford, UK | clinical, community-acquired infection | CA-MSSA | Jodi Lindsay. Department of Cellular and Molecular Medicine, St. George's, University of London, London, UK |
| GQ900406 | SAP048A | 27268 | NE 3809 | *S. aureus* | *abiK, cadXD, ser, sej, sed, sin*, ΔTn*552, repA, par* | RT2 | complete | 2006 | NE, USA | human clinical isolate, urine | | Kenneth Bayles: Department of Pathology and Microbiology, University of Nebraska Medical Center, Omaha, NE, USA |
| GQ900398 | pWBG744 | 27268 | WL6N | *S. aureus* | *abiK, cadXD, ser, sej, sed, sin*, ΔTn*552, repA, par* | RT2 | complete | 2001 | Remote Western Australia | screening | ST5-MSSA | Frances O'Brien. School of Biomedical Sciences, Curtin University of Technology, Perth, Western Australia |
| GQ900377 | SAP012A | 27267 | CDC31 | *S. aureus* | *abiK, cadXD, ser, sej, sed, sin*, ΔTn*552, repA, par* | RT2 | complete | 1995 | GA, USA | human clinical isolate | CA-MRSA | Jean Patel, Ainsley Nicholson, Brandi Limbago: Division of Healthcare Quality Promotion, Centers for Disease Control and Prevention, Atlanta, GA, USA |

| | | | | | | | | | | | | |
|---|---|---|---|---|---|---|---|---|---|---|---|---|
| GQ900380 | SAP015A | 27068 | CDC61 | *S. aureus* | *cadXD, sin*, ΔTn*552*, *aph,* sta, *aad,* bacitracin resistance, mac, ery, *rep, par* | RT3 | complete | 2002 | CA, USA | human clinical isolate | HA-MRSA | Jean Patel, Ainsley Nicholson, Brandi Limbago: Division of Healthcare Quality Promotion, Centers for Disease Control and Prevention, Atlanta, GA, USA |
| GQ900403 | SAP046A | 27068 | VET A6-001648 | *S. aureus* | *cadXD, sin*, ΔTn552, *aph,* sta, *aad,* bacitracin resistance, mac, ery, *repA, par* | RT3 | complete | 2005 | Clarke Co, GA, USA | canine abscess isolate | USA300 t008 ST8-ORSA-IV | Susan Sanchez: Department of Infectious Diseases, College of Veterinary Medicine, University of Georgia, Athens, GA, USA |
| GQ900409 | SAP050A | 27067 | NE 3874 | *S. aureus* | *cadXD, sin*, ΔTn*552*, *aph,* sta, *aad,* bacitracin resistance, mac, ery, *repA, par* | RT3 | complete | 2007 | NE, USA | human clinical isolate, wound | | Kenneth Bayles: Department of Pathology and Microbiology, University of Nebraska Medical Center, Omaha, NE, USA |
| GQ900452 | SAP105A | 26236 | SK933 | *S. epidermidis* | *cadCA, cadXD, mer, sin*, bacteriocin, *repA, par* | RT102 | complete | 1946 | Melbourne, Australia | human clinical isolate | | Ron Skurray, Neville Firth, Slade Jensen: School of Biological Sciences, University of Sydney, Sydney, AU |
| GQ900418 | SAP063A | 26016 | EMRSA-2 | *S. aureus* | *cadXD*, ΔTn*552*, *sin, repA, par* | RT34 | complete | 1987 | UK | clinical, hospital-acquired | HA-MRSA | Jodi Lindsay. Department of Cellular and |

| | | | | | | | | | | | | | |
|---|---|---|---|---|---|---|---|---|---|---|---|---|---|
| | | | | | | | | | | infection | | | Molecular Medicine, St. George's, University of London, London, UK |
| GQ900407 | SAP049A | 25022 | NE 3868 | *S. aureus* | *cadXD, sin*, ΔTn*552*, *aph,* sta, *aad,* bacitracin resistance, mac, ery, *repA, par* | RT5 (RT3-like) | complete | 2007 | NE, USA | human clinical isolate, eye | | | Kenneth Bayles: Department of Pathology and Microbiology, University of Nebraska Medical Center, Omaha, NE, USA |
| GQ900465 | SAP110A | 24711 | SK6536 | *S. epidermidis* | *aac,* antiseptic resistance, *bla,* metal transport, *repA, rep, par* | RT111 | complete | 2000 | Sydney, Australia | human clinical isolate | | | Ron Skurray, Neville Firth, Slade Jensen: School of Biological Sciences, University of Sydney, Sydney, AU |
| GQ900394 | pWBG752 | 24654 | WBG8404 | *S. aureus* | *ars, cadXD, pre, sin*, Tn*552*, bacteriocin, *repA, rep, par* | RT13 | complete | 1995 | Remote Western Australia | screening | ST45-MRSA-V (5C2) | | Frances O'Brien. School of Biomedical Sciences, Curtin University of Technology, Perth, Western Australia |
| GQ900458 | SAP108A | 24518 | SK85 | *S. epidermidis* | *aac, cadXD*, antiseptic resistance, mac, ery, *sin,* metal transport, *repA, rep, par* | RT108 | complete | 1980 | Melbourne, Australia | human clinical isolate | | | Ron Skurray, Neville Firth, Slade Jensen: School of Biological Sciences, University of Sydney, Sydney, AU |
| GQ900444 | pSK76 | 23983 | SK1373 | *S. aureus* | *ars, cadCA, mer, sin*, ΔTn*552, repA, par* | RT94 | complete | 1960 | Melbourne, Australia | human clinical isolate | | AsaCdHgPc | Ron Skurray, Neville Firth, Slade Jensen: School of Biological |

| | | | | | | | | | | | | | |
|---|---|---|---|---|---|---|---|---|---|---|---|---|---|
| | | | | | | | | | | | | | Sciences, University of Sydney, Sydney, AU |
| GQ900462 | pSK105 | 23263 | SK356 | *S. epidermidis* | *aacA-aphD,* IS256 x2, antiseptic resistance, *sin, repA, par* | RT109 | complete | 1980 | Melbourne, Australia | human clinical isolate | | QacAGmKm | Ron Skurray, Neville Firth, Slade Jensen: School of Biological Sciences, University of Sydney, Sydney, AU |
| GQ900410 | SAP051A | 23059 | NE 3883 | *S. aureus* | *cadXD, sin*, ΔTn*552*, *aph,* sta, *aad,* mac, ery, *repA, par* | RT8 (RT3-like) | complete | 2007 | NE, USA | human clinical isolate, wound | | | Kenneth Bayles: Department of Pathology and Microbiology, University of Nebraska Medical Center, Omaha, NE, USA |
| GQ900416 | SAP060A | 22987 | 502A | *S. aureus* | *abiK, cadXD, ser, sej, sed, sin, repA, par* | RT29 (RT2-like) | complete | pre-1960s | USA | not from infection | MSSA | | Jodi Lindsay. Department of Cellular and Molecular Medicine, St. George's, University of London, London, UK |
| GQ900446 | pSK62 | 22694 | SK1397 | *S. aureus* | *ars,* Tn*552*Δ, *cadCA, mer, sin* x2, *repA, par* | RT95 | complete | 1967 | Melbourne, Australia | human clinical isolate | | AsaCdHg | Ron Skurray, Neville Firth, Slade Jensen: School of Biological Sciences, University of Sydney, Sydney, AU |
| GQ900435 | p5753cA | 21806 | 5753c | *E. faecium* | *cad, van*, bacteriocin, iron transport | RT116 | complete | | | human clinical isolate | | | Jean Patel, Ainsley Nicholson, Brandi Limbago: Division of |

| | | | | | | | | | | | | |
|---|---|---|---|---|---|---|---|---|---|---|---|---|
| | | | | | | | | | | | | Healthcare Quality Promotion, Centers for Disease Control and Prevention, Atlanta, GA, USA |
| GQ900467 | pWBG763 | 20730 | WBG8366 | *S. aureus* | *cadXD, pre, sin*, Tn*552*, bacteriocin, *rep, par* | RT1 | complete | 1995 | Remote Western Australia | screening | ST78-MRSA-IVa (2B) | Frances O'Brien. School of Biomedical Sciences, Curtin University of Technology, Perth, Western Australia |
| GQ900397 | pWBG757 | 20730 | W17S | *S. aureus* | *cadXD, pre, sin*, Tn*552*, bacteriocin, *rep, par* | RT1 | complete | 1995 | Remote Western Australia | screening | ST93-MSSA | Frances O'Brien. School of Biomedical Sciences, Curtin University of Technology, Perth, Western Australia |
| GQ900425 | SAP073A | 20729 | 207 | *S. aureus* | *cadXD, pre, sin*, Tn*552*, bacteriocin, *rep, par* | RT1 | complete | 1999 | Oxford, UK | clinical, community-acquired infection | CA-MSSA | Jodi Lindsay. Department of Cellular and Molecular Medicine, St. George's, University of London, London, UK |
| GQ900413 | SAP053A | 20672 | NE 3890 | *S. aureus* | *cadXD, pre, sin*, Tn*552*, bacteriocin, *rep, par* | RT1 | complete | 2007 | NE, USA | human clinical isolate, wound | | Kenneth Bayles: Department of Pathology and Microbiology, University of Nebraska Medical Center, Omaha, NE, USA |

| | | | | | | | | | | | | | |
|---|---|---|---|---|---|---|---|---|---|---|---|---|---|
| GQ900392 | pWBG750 | 20653 | WBG8287 | *S. aureus* | *cadXD, pre, sin*, Tn*552*, bacteriocin, *rep, par* | RT1 | complete | 1995 | Royal Perth Hospital, WA | clinical, community-acquired infection | ST1-MRSA-IVa (2B) | | Frances O'Brien. School of Biomedical Sciences, Curtin University of Technology, Perth, Western Australia |
| GQ900424 | SAP072A | 20653 | 30067 | *S. aureus* | *cadXD, pre, sin*, Tn*552*, bacteriocin, *rep, par* | RT1 | complete | 2004 | UK | clinical, animal isolate | | | Jodi Lindsay. Department of Cellular and Molecular Medicine, St. George's, University of London, London, UK |
| GQ900414 | SAP055A | 20453 | 434 | *S. aureus* | *ars, cadXD, sin, repA, par* | RT24 | complete | 1999 | Oxford, UK | clinical, community-acquired infection | CA-MSSA | | Jodi Lindsay. Department of Cellular and Molecular Medicine, St. George's, University of London, London, UK |
| GQ900453 | SAP105B | 16775 | SK933 | *S. epidermidis* | *sin, repA, par* | ND | complete | 1946 | Melbourne, Australia | human clinical isolate | | | Ron Skurray, Neville Firth, Slade Jensen: School of Biological Sciences, University of Sydney, Sydney, AU |
| GQ900449 | SAP099B | 16428 | SK1271 | *S. aureus* | *ars, sin* x2, *repA*, Type Ib partitioning | ND | complete | 1951 | Melbourne, Australia | human clinical isolate | | AsaPcQacB | Ron Skurray, Neville Firth, Slade Jensen: School of Biological Sciences, University of Sydney, Sydney, AU |

| | | | | | | | | | | | | |
|---|---|---|---|---|---|---|---|---|---|---|---|---|
| GQ900429 | SAP077B | 13342 | 879R4RF | *S. aureus* | *traG,* possible ICE related to ICE6013 | ND | complete | 1982 | USA | not from infection | MSSA | Jodi Lindsay. Department of Cellular and Molecular Medicine, St. George's, University of London, London, UK |
| GQ900457 | SAP107B | 10445 | SK30 | *S. epidermidis* | antiseptic resistance, *mob, sin, repA, par* | ND | complete | 1980 | Melbourne, Australia | human clinical isolate | | Ron Skurray, Neville Firth, Slade Jensen: School of Biological Sciences, University of Sydney, Sydney, AU |
| GQ900376 | SAP008A | 9968 | 693-2 | *S. sp.* | *ars, mob, rep* | ND | complete | 2001 | GA, USA | chicken litter isolate | intI1-positive | Sobhan Nandi: Department of Microbiology, University of Georgia, Athens, GA, USA |
| GQ900455 | SAP106B | 8435 | SK939 | *S. epidermidis* | *mob* x2, *rep* | ND | complete | 1946 | Melbourne, Australia | human clinical isolate | | Ron Skurray, Neville Firth, Slade Jensen: School of Biological Sciences, University of Sydney, Sydney, AU |
| GQ900384 | SAP018B | 8226 | CDC25 | *S. sp. (CNS)* | *mob, rep* | ND | complete | 2001 | AR, USA | human clinical isolate | OxR | Jean Patel, Ainsley Nicholson, Brandi Limbago: Division of Healthcare Quality Promotion, Centers for Disease Control and Prevention, Atlanta, GA, USA |

| | | | | | | | | | | | | |
|---|---|---|---|---|---|---|---|---|---|---|---|---|
| GQ900466 | SAP110B | 6794 | SK6536 | *S. epidermidis* | *rep* remnant | ND | complete | 2000 | Sydney, Australia | human clinical isolate | | Ron Skurray, Neville Firth, Slade Jensen: School of Biological Sciences, University of Sydney, Sydney, AU |
| GQ900459 | SAP108B | 6206 | SK85 | *S. epidermidis* | *mob* x2, *rep* | ND | complete | 1980 | Melbourne, Australia | human clinical isolate | | Ron Skurray, Neville Firth, Slade Jensen: School of Biological Sciences, University of Sydney, Sydney, AU |
| GQ900463 | pSK103 | 4607 | SK356 | *S. epidermidis* | *mob, cat* | ND | complete | 1980 | Melbourne, Australia | human clinical isolate | Cm | Ron Skurray, Neville Firth, Slade Jensen: School of Biological Sciences, University of Sydney, Sydney, AU |
| GQ900436 | SAP084A | 4595 | SK1064 | *S. aureus* | *mob, cat* | ND | complete | 1953 | Melbourne, Australia | human clinical isolate | Cm | Ron Skurray, Neville Firth, Slade Jensen: School of Biological Sciences, University of Sydney, Sydney, AU |
| GQ900440 | SAP089A | 4595 | SK1404 | *S. aureus* | *mob, cat* | ND | complete | 1966 | Melbourne, Australia | human clinical isolate | Cm | Ron Skurray, Neville Firth, Slade Jensen: School of Biological Sciences, University of Sydney, Sydney, AU |
| GQ900460 | SAP108C | 4572 | SK85 | *S. epidermidis* | *mob* | ND | complete | 1980 | Melbourne, Australia | human clinical isolate | | Ron Skurray, Neville Firth, Slade Jensen: School of Biological |

| | | | | | | | | | | | | | |
|---|---|---|---|---|---|---|---|---|---|---|---|---|---|
| | | | | | | | | | | | | | Sciences, University of Sydney, Sydney, AU |
| GQ900417 | SAP060B | 4498 | 502A | *S. aureus* | *pre, tet* | ND | complete | pre-1960s | USA | not from infection | MSSA | | Jodi Lindsay. Department of Cellular and Molecular Medicine, St. George's, University of London, London, UK |
| GQ900437 | SAP085A | 4439 | SK1396 | *S. aureus* | *pre, tet* | ND | complete | 1965 | Melbourne, Australia | human clinical isolate | | Tc | Ron Skurray, Neville Firth, Slade Jensen: School of Biological Sciences, University of Sydney, Sydney, AU |
| GQ900441 | SAP093A | 4439 | SK1529 | *S. aureus* | *pre, tet* | ND | complete | 1965 | Melbourne, Australia | human clinical isolate | | Tc | Ron Skurray, Neville Firth, Slade Jensen: School of Biological Sciences, University of Sydney, Sydney, AU |
| GQ900443 | SAP094A | 4439 | SK449 | *S. aureus* | *pre, tet* | ND | complete | 1981 | Melbourne, Australia | human clinical isolate | | Tc | Ron Skurray, Neville Firth, Slade Jensen: School of Biological Sciences, University of Sydney, Sydney, AU |
| GQ900445 | SAP095B | 4439 | SK1373 | *S. aureus* | *pre, tet* | ND | complete | 1960 | Melbourne, Australia | human clinical isolate | | Tc | Ron Skurray, Neville Firth, Slade Jensen: School of Biological Sciences, University of Sydney, Sydney, |

| | | | | | | | | | | | | | |
|---|---|---|---|---|---|---|---|---|---|---|---|---|---|
| | | | | | | | | | | | | | AU |
| GQ900438 | SAP085B | 3961 | SK1396 | *S. aureus* | *pre,* mac-linc-str-ery | ND | complete | 1965 | Melbourne, Australia | human clinical isolate | | AsaCdHg | Ron Skurray, Neville Firth, Slade Jensen: School of Biological Sciences, University of Sydney, Sydney, AU |
| GQ900404 | SAP046B | 3125 | VET A6-001648 | *S. aureus* | | ND | complete | 2005 | Clarke Co, GA, USA | canine abscess isolate | mec type IVh | | Susan Sanchez: Department of Infectious Diseases, College of Veterinary Medicine, University of Georgia, Athens, GA, USA |
| GQ900408 | SAP049B | 3125 | NE 3868 | *S. aureus* | | ND | complete | 2007 | NE, USA | human clinical isolate, eye | | | Kenneth Bayles: Department of Pathology and Microbiology, University of Nebraska Medical Center, Omaha, NE, USA |
| GQ900411 | SAP051B | 3125 | NE 3883 | *S. aureus* | | ND | complete | 2007 | NE, USA | human clinical isolate, wound | | | Kenneth Bayles: Department of Pathology and Microbiology, University of Nebraska Medical Center, Omaha, NE, USA |
| GQ900450 | SAP104A | 3011 | SK6575 | *S. aureus* | | ND | complete | 1998 | Bangkok, | human | | | Ron Skurray, Neville |

| | | | | | | | | | | | | | |
|---|---|---|---|---|---|---|---|---|---|---|---|---|---|
| | | | | | | | | | Thailand | clinical isolate | | | Firth, Slade Jensen: School of Biological Sciences, University of Sydney, Sydney, AU |
| GQ900423 | SAP070A | 3011 | S54 | *S. aureus* | | ND | complete | 2003 | London, UK | clinical, hospital-acquired infection | HA-MRSA | | Jodi Lindsay. Department of Cellular and Molecular Medicine, St. George's, University of London, London, UK |
| GQ900420 | SAP065A | 2908 | EMRSA-10 | *S. aureus* | *cat* | ND | complete | 1987 | UK | clinical, hospital-acquired infection | HA-MRSA | | Jodi Lindsay. Department of Cellular and Molecular Medicine, St. George's, University of London, London, UK |
| GQ900393 | pWBG751 | 2473 | WBG8287 | *S. aureus* | mac-linc-str-ery | ND | complete | 1995 | Royal Perth Hospital, WA | clinical, community-acquired infection | ST1-MRSA-IVa (2B) | | Frances O'Brien. School of Biomedical Sciences, Curtin University of Technology, Perth, Western Australia |
| GQ900461 | SAP108D | 2422 | SK85 | *S. epidermidis* | | ND | complete | 1980 | Melbourne, Australia | human clinical isolate | | | Ron Skurray, Neville Firth, Slade Jensen: School of Biological Sciences, University of Sydney, Sydney, AU |
| GQ900464 | pSK108 | 2418 | SK356 | *S. epidermidis* | *smr,* partial *qacC* | ND | complete | 1980 | Melbourne, Australia | human clinical | | QacC | Ron Skurray, Neville Firth, Slade Jensen: |

| | | | | | | | | | | | | | |
|---|---|---|---|---|---|---|---|---|---|---|---|---|---|
| | | | | | | | | | | isolate | | | School of Biological Sciences, University of Sydney, Sydney, AU |
| GQ900431 | SAP078B | 2415 | A78R1 | *S. aureus* | mac-linc-str-ery | ND | complete | 2007 | UK | not from infection | MRSA | | Jodi Lindsay. Department of Cellular and Molecular Medicine, St. George's, University of London, London, UK |
| GQ900468 | pWBG764 | 2397 | WBG8366 | *S. aureus* | | ND | complete | 1995 | Remote Western Australia | screening | ST78-MRSA-IVa (2B) | | Frances O'Brien. School of Biomedical Sciences, Curtin University of Technology, Perth, Western Australia |
| GQ900439 | SAP087A | 2396 | SK1020 | *S. aureus* | | ND | complete | 1947 | Melbourne, Australia | human clinical isolate | | AsaCdHg | Ron Skurray, Neville Firth, Slade Jensen: School of Biological Sciences, University of Sydney, Sydney, AU |
| GQ900396 | pWBG754 | 2241 | WA8 | *S. aureus* | multidrug efflux | ND | complete | 2003 | Royal Perth Hospital, WA | clinical, community-acquired infection | ST75-MRSA-IVa (2B) | | Frances O'Brien. School of Biomedical Sciences, Curtin University of Technology, Perth, Western Australia |
| GQ900451 | SAP104B | 1552 | SK6575 | *S. aureus* | | ND | complete | 1998 | Bangkok, Thailand | human clinical isolate | | | Ron Skurray, Neville Firth, Slade Jensen: School of Biological Sciences, University |

| | | | | | | | | | | | | | |
|---|---|---|---|---|---|---|---|---|---|---|---|---|---|
| | | | | | | | | | | | | | of Sydney, Sydney, AU |
| GQ900442 | SAP093B | 1290 | SK1529 | *S. aureus* | | ND | complete | 1965 | Melbourne, Australia | human clinical isolate | | AsaCdHgPc | Ron Skurray, Neville Firth, Slade Jensen: School of Biological Sciences, University of Sydney, Sydney, AU |
| GQ900497 | SAP103A | 29258 | SK6562 | *S. aureus* | *cadCA, sin, tet*, Tn*552*, *pre* | RT100 | partial, single gap ~20 bp | 2000 | Sydney, Australia | human clinical isolate | | | Ron Skurray, Neville Firth, Slade Jensen: School of Biological Sciences, University of Sydney, Sydney, AU |
| GQ900495 | SAP101A | 32163 | SK6522 | *S. aureus* | *cadCA, cadXD, mer, sin*, antiseptic resistance, IS256, ΔTn*552* | RT39 | partial, single gap ~20 bp | 2000 | Sydney, Australia | human clinical isolate | | | Ron Skurray, Neville Firth, Slade Jensen: School of Biological Sciences, University of Sydney, Sydney, AU |
| GQ900493 | pSK57 | 31164 | SK449 | *S. aureus* | *cadCA, cadXD, mer, sin*, antiseptic resistance, ΔTn*552* | RT93 | partial, single gap ~20 bp | 1981 | Melbourne, Australia | human clinical isolate | | CdHgPcQacA | Ron Skurray, Neville Firth, Slade Jensen: School of Biological Sciences, University of Sydney, Sydney, AU |
| GQ900492 | pSK80 | 31314 | SK1529 | *S. aureus* | *ars, cadCA, cadXD, mer, sin,* ΔTn*552* | RT92 | partial, single gap ~20 bp | 1965 | Melbourne, Australia | human clinical isolate | | AsaCdHgPc | Ron Skurray, Neville Firth, Slade Jensen: School of Biological Sciences, University of Sydney, Sydney, AU |

| | | | | | | | | | | | | | |
|---|---|---|---|---|---|---|---|---|---|---|---|---|---|
| GQ900491 | pSK23 | 41993 | SK654 | *S. aureus* | *bin* x2, *bla, cadCA, cadXD, mer, sin, aacA-aphD*, antiseptic resistance, IS256 x2 | RT91 | partial, single gap ~20 bp | 1981 | Hobart, Australia | human clinical isolate | | CdHgQacBGmKm | Ron Skurray, Neville Firth, Slade Jensen: School of Biological Sciences, University of Sydney, Sydney, AU |
| GQ900490 | pSK21 | 37046 | SK413 | *S. aureus* | *cadCA, cadXD, mer, sin*, antiseptic resistance, ΔTn*552* | RT90 | partial, single gap ~20 bp | 1981 | Melbourne, Australia | human clinical isolate | | CdHgPcQacB | Ron Skurray, Neville Firth, Slade Jensen: School of Biological Sciences, University of Sydney, Sydney, AU |
| GQ900489 | pSK79 | 35850 | SK1215 | *S. aureus* | *ars, cadCA, cadXD, mer, sin* x2, ΔTn*552* | RT87 | partial, single gap ~20 bp | 1953 | Melbourne, Australia | human clinical isolate | | AsaCdHgPc | Ron Skurray, Neville Firth, Slade Jensen: School of Biological Sciences, University of Sydney, Sydney, AU |
| GQ900488 | pSK59 | 29570 | SK1396 | *S. aureus* | *ars, cadCA, cadXD, mer, sin* x2, Tn*552*Δ | RT86 | partial, single gap ~20 bp | 1965 | Melbourne, Australia | human clinical isolate | | AsaCdHg | Ron Skurray, Neville Firth, Slade Jensen: School of Biological Sciences, University of Sydney, Sydney, AU |
| GQ900484 | SAP070B | 29531 | S54 | *S. aureus* | antiseptic resistance, *cadCA, cadXD, mer, sin*, Tn*552*Δ | RT42 | partial, single gap ~20 bp | 2003 | London, UK | clinical, hospital-acquired infection | HA-MRSA | | Jodi Lindsay. Department of Cellular and Molecular Medicine, St. George's, University of London, London, UK |
| GQ900482 | SAP066A | 32122 | EMRSA-17 | *S. aureus* | *cadCA, cadXD, mer, sin*, antiseptic resistance, | RT39 | partial, single gap ~20 bp | 2000 | UK | clinical, hospital- | HA-MRSA | | Jodi Lindsay. Department of |

| | | | | | | | | | | | | |
|---|---|---|---|---|---|---|---|---|---|---|---|---|
| | | | | | ΔTn*552*, IS256 | | | | | acquired infection | | Cellular and Molecular Medicine, St. George's, University of London, London, UK |
| GQ900481 | SAP065B | 26750 | EMRSA-10 | *S. aureus* | *ars, cadCA, cadXD, sin*, Tn*552*, *bin* | RT36 | partial, single gap ~20 bp | 1987 | UK | clinical, hospital-acquired infection | HA-MRSA | Jodi Lindsay. Department of Cellular and Molecular Medicine, St. George's, University of London, London, UK |
| GQ900480 | SAP059A | 26243 | 3172 | *S. aureus* | *cadXD, sin, sdrE, pls* | RT28 | partial, single gap ~20 bp | 1999 | Oxford, UK | not from infection | MSSA | Jodi Lindsay. Department of Cellular and Molecular Medicine, St. George's, University of London, London, UK |
| GQ900475 | pWBG762 | 54023 | K102N | *S. aureus* | *cadXD, pre, se* x2, *sin,* Tn*552*, bacteriocin | RT22 | partial, single gap ~20 bp | 1995 | Remote Western Australia | screening | ST508-MSSA | Frances O'Brien. School of Biomedical Sciences, Curtin University of Technology, Perth, Western Australia |
| GQ900472 | pWBG756 | 24456 | WSPP | *S. aureus* | *cadXD, sin*, Tn*552* | RT18 | partial, single gap ~20 bp | 2002 | Royal Perth Hospital, WA | clinical, community-acquired infection | ST30-MRSA-IV | Frances O'Brien. School of Biomedical Sciences, Curtin University of Technology, Perth, Western Australia |

| | | | | | | | | | | | | | |
|---|---|---|---|---|---|---|---|---|---|---|---|---|---|
| GQ900471 | pWBG755 | 25607 | WA8 | *S. aureus* | *cadXD, sin*, $\Delta$Tn*552* | RT117 | partial, single gap ~20 bp | 2003 | Royal Perth Hospital, WA | clinical, community-acquired infection | ST75-MRSA-IVa (2B) | | Frances O'Brien. School of Biomedical Sciences, Curtin University of Technology, Perth, Western Australia |
| GQ900469 | SAP024A | 47352 | CDC19 | *S. epidermidis* | *cadXD, mob, sin* | RT48 | partial, single gap ~20 bp | 1998 | WI, USA | human clinical isolate | OxR | | Jean Patel, Ainsley Nicholson, Brandi Limbago: Division of Healthcare Quality Promotion, Centers for Disease Control and Prevention, Atlanta, GA, USA |
| GQ915272 | pSK53 | 24475 | SK1700 | *S. aureus* | Tn*552*, *tra1,* possible ICE | ND | partial | 1975 | Melbourne, Australia | human clinical isolate | | CdHgPc | Ron Skurray, Neville Firth, Slade Jensen: School of Biological Sciences, University of Sydney, Sydney, AU |
| GQ915271 | phage SAP090B | 26877 | SK1700 | *S. aureus* | only phage genes | ND | partial | 1975 | Melbourne, Australia | human clinical isolate | | | Ron Skurray, Neville Firth, Slade Jensen: School of Biological Sciences, University of Sydney, Sydney, AU |
| GQ915270 | pSK53 | 38886 | SK1700 | *S. aureus* | *cadCA, cadXD, mer, qacA, sin*, $\Delta$Tn*552* | ND | partial | 1975 | Melbourne, Australia | human clinical isolate | | CdHgPc | Ron Skurray, Neville Firth, Slade Jensen: School of Biological Sciences, University of Sydney, Sydney, AU |

| | | | | | | | | | | | | | |
|---|---|---|---|---|---|---|---|---|---|---|---|---|---|
| GQ915269 | pSK73 | 39907 | SK1404 | *S. aureus* | mac-linc-str-ery, *aad* | RT88 | partial | 1966 | Melbourne, Australia | human clinical isolate | | AsaCdHg | Ron Skurray, Neville Firth, Slade Jensen: School of Biological Sciences, University of Sydney, Sydney, AU |
| GQ915268 | pSK64 | 34784 | SK1020 | *S. aureus* | *ars, cadCA, cadXD, mer, sin*, Tn*552*Δ | RT87 | partial | 1947 | Melbourne, Australia | human clinical isolate | | AsaCdHg | Ron Skurray, Neville Firth, Slade Jensen: School of Biological Sciences, University of Sydney, Sydney, AU |
| GQ915267 | pSK60 | 35175 | SK1011 | *S. aureus* | *ars, cadCA, cadXD, mer, sin*, ΔTn*552* | RT87 | partial | 1947 | Melbourne, Australia | human clinical isolate | | AsaCdHgPc | Ron Skurray, Neville Firth, Slade Jensen: School of Biological Sciences, University of Sydney, Sydney, AU |
| GQ915266 | pSK74 | 35327 | SK1064 | *S. aureus* | *ars, cadCA, cadXD, mer,* Tn*552, bin, blaI, blaR1* | RT85 | partial | 1953 | Melbourne, Australia | human clinical isolate | | AsaCdHgPc | Ron Skurray, Neville Firth, Slade Jensen: School of Biological Sciences, University of Sydney, Sydney, AU |
| GQ915265 | pWBG748 | 44964 | WBG8381 | *S. aureus* | *bla, tra* (incomplete) | RT9 | partial | 1995 | Remote Western Australia | screening | ST5-MRSA-IVa (2B) | | Frances O'Brien. School of Biomedical Sciences, Curtin University of Technology, Perth, Western Australia |
| GQ915264 | SAP015B | 7069 | CDC61 | *S. aureus* | | RT50 | partial | 2002 | CA, USA | human clinical isolate | HA-MRSA | | Jean Patel, Ainsley Nicholson, Brandi Limbago: Division of |

| | | | | | | | | | | | | | |
|---|---|---|---|---|---|---|---|---|---|---|---|---|---|
| | | | | | | | | | | | | | Healthcare Quality Promotion, Centers for Disease Control and Prevention, Atlanta, GA, USA |
| GQ915263 | SAP015B | 12605 | CDC61 | *S. aureus* | *tra* (incomplete), possible ICE | RT50 | partial | 2002 | CA, USA | human clinical isolate | HA-MRSA | | Jean Patel, Ainsley Nicholson, Brandi Limbago: Division of Healthcare Quality Promotion, Centers for Disease Control and Prevention, Atlanta, GA, USA |
| GQ900515 | SAP102B | 1858 | SK6523 | *S. aureus* | | ND | partial | 2000 | Sydney, Australia | human clinical isolate | | | Ron Skurray, Neville Firth, Slade Jensen: School of Biological Sciences, University of Sydney, Sydney, AU |
| GQ900514 | pSK17 | 7929 | SK707 | *S. aureus* | *sin, aacA-aphD*, antiseptic resistance, IS256 | RT96 | partial | 1981 | Melbourne, Australia | human clinical isolate | | PcQacAGmKmTp | Ron Skurray, Neville Firth, Slade Jensen: School of Biological Sciences, University of Sydney, Sydney, AU |
| GQ900513 | pSK17 | 9968 | SK707 | *S. aureus* | | RT96 | partial | 1981 | Melbourne, Australia | human clinical isolate | | PcQacAGmKmTp | Ron Skurray, Neville Firth, Slade Jensen: School of Biological Sciences, University of Sydney, Sydney, AU |
| GQ900512 | phage SAP090D | 10726 | SK1700 | *S. aureus* | only phage genes | ND | partial | 1975 | Melbourne, Australia | human clinical | | | Ron Skurray, Neville Firth, Slade Jensen: |

| | | | | | | | | | | | | |
|---|---|---|---|---|---|---|---|---|---|---|---|---|
| | | | | | | | | | | isolate | | School of Biological Sciences, University of Sydney, Sydney, AU |
| GQ900511 | SAP076B | 1979 | PM86 | *S. aureus* | | ND | partial | 1999 | SGH, London, UK | not from infection | HA-MRSA | Jodi Lindsay. Department of Cellular and Molecular Medicine, St. George's, University of London, London, UK |
| GQ900510 | SAP067B | 1415 | PM62 | *S. aureus* | | ND | partial | 1999 | SGH, London, UK | clinical, hospital-acquired infection | HA-MRSA | Jodi Lindsay. Department of Cellular and Molecular Medicine, St. George's, University of London, London, UK |
| GQ900509 | SAP062D | 4764 | EMRSA-1 | *S. aureus* | antiseptic resistance, IS256 | RT33 | partial | 1984 | UK | clinical, hospital-acquired infection | HA-MRSA | Jodi Lindsay. Department of Cellular and Molecular Medicine, St. George's, University of London, London, UK |
| GQ900508 | SAP062C | 6516 | EMRSA-1 | *S. aureus* | *mob,* IS256 | RT33 | partial | 1984 | UK | clinical, hospital-acquired infection | HA-MRSA | Jodi Lindsay. Department of Cellular and Molecular Medicine, St. George's, University of London, London, UK |

| | | | | | | | | | | | | |
|---|---|---|---|---|---|---|---|---|---|---|---|---|
| GQ900507 | SAP062B | 9840 | EMRSA-1 | *S. aureus* | Tn*552*, *sin* | RT33 | partial | 1984 | UK | clinical, hospital-acquired infection | HA-MRSA | Jodi Lindsay. Department of Cellular and Molecular Medicine, St. George's, University of London, London, UK |
| GQ900506 | SAP062A | 10083 | EMRSA-1 | *S. aureus* | | RT33 | partial | 1984 | UK | clinical, hospital-acquired infection | HA-MRSA | Jodi Lindsay. Department of Cellular and Molecular Medicine, St. George's, University of London, London, UK |
| GQ900505 | SAP024B | 14823 | CDC19 | *S. epidermidis* | *mob, dfr* | ND | partial | 1998 | WI, USA | human clinical isolate | OxR | Jean Patel, Ainsley Nicholson, Brandi Limbago: Division of Healthcare Quality Promotion, Centers for Disease Control and Prevention, Atlanta, GA, USA |
| GQ900504 | SAP015B | 2712 | CDC61 | *S. aureus* | | RT50 | partial | 2002 | CA, USA | human clinical isolate | HA-MRSA | Jean Patel, Ainsley Nicholson, Brandi Limbago: Division of Healthcare Quality Promotion, Centers for Disease Control and Prevention, Atlanta, GA, USA |
| GQ900503 | SAP015B | 2993 | CDC61 | *S. aureus* | | RT50 | partial | 2002 | CA, USA | human clinical isolate | HA-MRSA | Jean Patel, Ainsley Nicholson, Brandi Limbago: Division of |

| | | | | | | | | | | | | |
|---|---|---|---|---|---|---|---|---|---|---|---|---|
| | | | | | | | | | | | | Healthcare Quality Promotion, Centers for Disease Control and Prevention, Atlanta, GA, USA |
| GQ900502 | SAP015B | 6774 | CDC61 | *S. aureus* | lincosamide resistance | RT50 | partial | 2002 | CA, USA | human clinical isolate | HA-MRSA | Jean Patel, Ainsley Nicholson, Brandi Limbago: Division of Healthcare Quality Promotion, Centers for Disease Control and Prevention, Atlanta, GA, USA |
| GQ900501 | SAP015B | 9066 | CDC61 | *S. aureus* | *res* | RT50 | partial | 2002 | CA, USA | human clinical isolate | HA-MRSA | Jean Patel, Ainsley Nicholson, Brandi Limbago: Division of Healthcare Quality Promotion, Centers for Disease Control and Prevention, Atlanta, GA, USA |
| GQ900500 | SAP015B | 21860 | CDC61 | *S. aureus* | *tra, aacA-aphD*, kan, IS256 x2 | RT50 | partial | 2002 | CA, USA | human clinical isolate | HA-MRSA | Jean Patel, Ainsley Nicholson, Brandi Limbago: Division of Healthcare Quality Promotion, Centers for Disease Control and Prevention, Atlanta, GA, USA |
| GQ900499 | pSK101 | 4256 | SK356 | *S. epidermidis* | *tet* | ND | partial | 1980 | Melbourne, Australia | human clinical isolate | | Ron Skurray, Neville Firth, Slade Jensen: School of Biological Sciences, University |

| | | | | | | | | | | | | | |
|---|---|---|---|---|---|---|---|---|---|---|---|---|---|
| | | | | | | | | | | | | | of Sydney, Sydney, AU |
| GQ900498 | SAP104C | 29343 | SK6575 | *S. aureus* | *bin, cadCA, cadXD, mer, sin*, antiseptic resistance, IS256 | RT101 | partial | 1998 | Bangkok, Thailand | human clinical isolate | | | Ron Skurray, Neville Firth, Slade Jensen: School of Biological Sciences, University of Sydney, Sydney, AU |
| GQ900496 | SAP102A | 36082 | SK6523 | *S. aureus* | *ars, bin, cadCA, sin, pre*, biofilm-associated protein, metal transport | RT99 | partial | 2000 | Sydney, Australia | human clinical isolate | | | Ron Skurray, Neville Firth, Slade Jensen: School of Biological Sciences, University of Sydney, Sydney, AU |
| GQ900494 | pSK77 | 27694 | SK1499 | *S. aureus* | *sin, dfr, aacA-aphD*, antiseptic resistance, IS256 x2 | RT96 | partial | 1965 | Melbourne, Australia | human clinical isolate | | CdPc | Ron Skurray, Neville Firth, Slade Jensen: School of Biological Sciences, University of Sydney, Sydney, AU |
| GQ900487 | p5753cB | 56689 | 5753c | *E. faecium* | *sin, aac, aph, aad*, sta, mac-linc-str, bacteriocin, iron transport, IS256, *mob, tet* | RT116 | partial | | | human clinical isolate | | | Jean Patel, Ainsley Nicholson, Brandi Limbago: Division of Healthcare Quality Promotion, Centers for Disease Control and Prevention, Atlanta, GA, USA |
| GQ900486 | SAP075A | 37050 | B111 | *S. aureus* | *ars, bin, cadCA, pre, sin*, metal transport, biofilm-associated protein | RT30 | partial | 1992 | London, UK | | MSSA | | Jodi Lindsay. Department of Cellular and Molecular Medicine, St. George's, |

| | | | | | | | | | | | | |
|---|---|---|---|---|---|---|---|---|---|---|---|---|
| | | | | | | | | | | | | University of London, London, UK |
| GQ900485 | SAP071A | 26883 | C00704 | *S. aureus* | *pls, abiK, cadXD, sin*, ΔTn*552* | RT20 | partial | 2004 | UK | clinical, animal isolate | | Jodi Lindsay. Department of Cellular and Molecular Medicine, St. George's, University of London, London, UK |
| GQ900483 | SAP067A | 37206 | PM62 | *S. aureus* | *ars, bin, cadCA, pre, sin*, metal transport, biofilm-associated protein | RT40 | partial | 1999 | SGH, London, UK | clinical, hospital-acquired infection | HA-MRSA | Jodi Lindsay. Department of Cellular and Molecular Medicine, St. George's, University of London, London, UK |
| GQ900479 | SAP058A | 22439 | 3134 | *S. aureus* | *abiK, cadXD, sin, pls* | RT27 | partial | 1999 | Oxford, UK | not from infection | MSSA | Jodi Lindsay. Department of Cellular and Molecular Medicine, St. George's, University of London, London, UK |
| GQ900478 | SAP056A | 27128 | 3041 | *S. aureus* | *cadXD*, biofilm-associated protein | RT25 | partial | 1999 | Oxford, UK | not from infection | MSSA | Jodi Lindsay. Department of Cellular and Molecular Medicine, St. George's, University of London, London, UK |

| | | | | | | | | | | | | |
|---|---|---|---|---|---|---|---|---|---|---|---|---|
| GQ900477 | SAP054A | 37475 | 49 | *S. aureus* | *ars, bin, cadCA, pre, sin*, metal transport | RT23 | partial | 1999 | Oxford, UK | clinical, community-acquired infection | CA-MSSA | Jodi Lindsay. Department of Cellular and Molecular Medicine, St. George's, University of London, London, UK |
| GQ900476 | SAP044A | 36242 | 693-7 | *S. sp. (CNS)* | *cadXD, pre*, bacitracin resistance | RT59 | partial | 2001 | GA, USA | chicken litter isolate | intI1-positive | Sobhan Nandi: Department of Microbiology, University of Georgia, Athens, GA, USA |
| GQ900474 | pWBG761 | 26838 | C33S | *S. aureus* | *abiK, cadXD, sin, pls*, ΔTn*552* | RT20 | partial | 1995 | Remote Western Australia | screening | ST12-MSSA | Frances O'Brien. School of Biomedical Sciences, Curtin University of Technology, Perth, Western Australia |
| GQ900473 | pWBG760 | 4412 | C57S | *S. aureus* | | ND | partial | 1995 | Remote Western Australia | screening | ST20-MSSA | Frances O'Brien. School of Biomedical Sciences, Curtin University of Technology, Perth, Western Australia |
| GQ900470 | SAP025A | 35220 | CDC2 | *S. epidermidis* | *bin, sin, sdrE* | RT57 | partial | 1996 | SC, USA | human clinical isolate | OxS | Jean Patel, Ainsley Nicholson, Brandi Limbago: Division of Healthcare Quality Promotion, Centers for Disease Control and Prevention, Atlanta, GA, USA |

[a] Complete sequences are sorted first by descending size, then partial sequences with a single gap by size, then partial sequences by descending accession number

[b] Selected genotype highlights, not an all-inclusive list of genes:

Tn*552* = *p271*, *p480* (or Tn*552* transposase), *bin, blaI, blaR1, blaZ*
ΔTn*552* = *bin, blaI, blaR1, blaZ*
Tn*552*Δ = *p271, p480* (or Tn*552* transposase), *bin*
IS256 = IS256 transposase
*aac* = aminoglycoside N-acetyltranferase
*aacA-aphD* = aminoglycoside resistance
*aad* = aminoglycoside 6-adenylyltransferase
*aph* = Aminoglycoside 3'-phosphotransferase (Kanamycin kinase, type III) (Neomycin-kanamycin phosphotransferase type III)
asp/sec = secretion of virulence glycoproteins
*bla* = *blaI, blaR1, blaZ*
ble = bleomycin resistance
*cad* = cadmium resistance: cadD, cadX, cadC, cadA
*cat* = chloramphenicol ecetyltransferase
*dfr* = dihydrofolate reductase
ery = erythromycin resistance
*etb* = exfoliatin B
exo = exoenzyme C3 precursor
kan = kanamycin resistance
mac = macrolide resistance
mac-linc-str = rRNA adenine N-6-methyltransferase (Macrolide-lincosamide-streptogramin B resistance protein)
*mob* = mobilization gene(s)
*par* = pSK1-type plasmid partitioning; only identified for complete sequences
*pls* = antiadhesin Pls, binding to squamous nasal epithelial cells
*pre* = plasmid recombination enzyme, relaxase
*qacA* or *qacC* = antiseptic resistanc
*repA* = N-type replication initiation; only identified for complete sequences
*rep* = pSK639-type replication; only identified for complete sequences
*sdrE* = adhesin protein
*se* = staphylococcal enterotoxin
*smr* = multidrug efflux protein
sta = streptothricin acetyltransferase
*tcaA* = teicoplanin resistance
*tet* = tetracycline resistance
*tra* = conjugative transfer loci
Type Ib and II partitioning only identified for complete sequences

[c] OxR/S = oxacillin resistant/sensitive

CA = community-associated
HA = hospital-associated
MRSA = methicillin resistant *Staphylococcus aureus*
MSSA = methicillin sensitive *Staphylococcus aureus*
ST = MLST sequence type

[d] Previously determined plasmid phenotypes
Asa = arsenate/arsenite
Cd = cadmium
Cm = chloramphenicol
Gm = gentamycin
Hg = mercury
Km = kanamycin
Pc = penicillin
QacA/B/C = quaternary ammonium antiseptic
Tc = tetracycline
Tp = trimethoprim

**Table S3 Large plasmid content of newly screened *Staphylococcus* strains**

| | Number of strains (%) |
|---|---|
| Total newly examined[a] | 247 |
| 1 or more plasmids >20kb | 194 (78.5%) |
| Only <20kb plasmids | 25 (10.1%) |
| No plasmids | 28 (11.3%) |

[a] These strains were not previously examined for plasmid content
