# Supplementary material for: Major Families of Multiresistant Plasmids from Geographically and Epidemiologically Diverse Staphylococci
Source: G3 (Bethesda). 2011 Dec 1;1(7):581–91. doi: 10.1534/g3.111.000760 (PMC3276174; doi:10.1534/g3.111.000760)
Supplement: Supporting Information [file supp_1.7.581_TableS1.pdf]

Table S1 Strain collections used in this work

Strain collection	Source	Description	Number screened
NARSA[a]	NARSA[a], US	Human clinical	10
CDC[b]	Centers for Disease Control and Prevention, US	Human clinical	64[b]
Veterinary	University of Georgia, GA, US	Companion animal	14
Poultry	University of Georgia, GA, US	Poultry litter	8
Nebraska	University of Nebraska Medical Center, NE, US	Human clinical	51
Western Australia	Curtin University of Technology, Perth, Western Australia	Human clinical	35
United Kingdom	University of London, London, UK	Human and animal clinical, screenings and infections	65
Historic	University of Sydney, Sydney, AU	Human clinical	29
**Total**			**280**

[a] Network on Antimicrobial Resistance in *Staphylococcus aureus* (NARSA) (http://www.narsa.net)
[b] There were four additional transconjugant strains with plasmids from human clinical strains in this collection (McDougal *et al*. 2008).
