# Supplementary material for: Major Families of Multiresistant Plasmids from Geographically and Epidemiologically Diverse Staphylococci
Source: G3 (Bethesda). 2011 Dec 1;1(7):581–91. doi: 10.1534/g3.111.000760 (PMC3276174; doi:10.1534/g3.111.000760)
Supplement: Supporting Information [file supp_1.7.581_TableS3.pdf]

Table S3 Large plasmid content of newly screened *Staphylococcus* strains

	Number of strains (%)
Total newly examined[a]	247
1 or more plasmids >20kb	194 (78.5%)
Only <20kb plasmids	25 (10.1%)
No plasmids	28 (11.3%)

[a] These strains were not previously examined for plasmid content
